# Fullerol Nanocatalysis and Trimodal Surface Plasmon Resonance for the Determination of Isocarbophos

**DOI:** 10.3389/fchem.2020.00673

**Published:** 2020-08-14

**Authors:** Huixiang Ouyang, Aihui Liang, Zhiliang Jiang

**Affiliations:** ^1^Guangxi Colleges and Universities Key Laboratory of Regional Ecological Environment Analysis and Pollution Control of West Guangxi, College of Chemistry and Environment Engineering, Baise University, Baise, China; ^2^Key Laboratory of Ecology of Rare and Endangered Species and Environmental Protection of Ministry Education, Guangxi Key Laboratory of Environmental Pollution Control Theory and Technology, Guangxi Normal University, Guilin, China

**Keywords:** isocarbophos, aptamer, fullerol nanocatalysis, SERS, RRS, Abs

## Abstract

Fullerol (C_60_OH) has been shown to catalyze the trisodium citrate (TSC)–silver nitrate reaction to generate Ag nanoparticles (AgNPs). These AgNPs exhibit significant nanoplasmic surface-enhanced Raman scattering (SERS), resonance Rayleigh scattering (RRS), and absorption (Abs). When an aptamer (Apt) adsorbs on the C_60_OH surface, catalysis is inhibited, and the intensities of SERS, RRS, and Abs decrease. In the presence of isocarbophos (IPS), Apt forms a stable complex (Apt-IPS) and releases C_60_OH. As a result, SERS, RRS, and Abs intensities increase with increasing IPS concentration. Accordingly, a new SERS, RRS, and Abs trimodal method using Apt-labeled fullerol was established for the determination of IPS. Of the three spectral methods, SERS was the most sensitive, while the Abs method was the most cost-effective.

## Introduction

Surface plasmon resonance (SPR) is an optical phenomenon caused by the oscillation of free electrons in a metal surface layer produced by incident light (Jackman et al., 2017). Because of their exponentially larger surface areas, metal nanoparticles exhibit enhanced SPR and produce a more sensitive response. With the development of nanomaterial fabrication technologies, SPR has been used increasingly for the analysis of metal nanomaterials (Ye et al., 2016; Ouyang et al., 2017). Surface-enhanced Raman spectroscopy (SERS), in which the SPR effect is amplified by substances adsorbed on the nanoparticle surface, is an increasingly popular direct application of nanoscale plasma detection (Alvarez-Puebla and Liz-Marzan, 2012). Nanomaterials, especially those comprising noble metals and carbon nanomaterials, have novel spectral, electric, magnetic, thermal, and chemical properties (Gao et al., 2007; Kotov, 2010; Wei and Wang, 2013). Because noble metal nanomaterials, such as AuNPs and AgNPs, possess both catalytic activity and SERS activity, they have drawn attention (Jiang et al., 2008, 2010a,b; Liang et al., 2011, 2015; Yao et al., 2013). Carbon-based nanomaterials have abundant conjugated π bonds (C=C), which are characterized by high electron density, delocalization, and electron transfer ability (Krätschmer et al., 1990; Zhang et al., 2011; Zhao et al., 2016; Zhou et al., 2016; Justino et al., 2017) and have potential as green catalysts. Fullerene is of particular interest as a promising carbon nanomaterial, and it has been widely used in solar energy conversion materials and catalysis (Zhao et al., 2016; Cai et al., 2017) since it was successfully prepared. C_60_ is stable and possesses good electron-transfer ability, due to the highly delocalized conjugated system consisting of 30 C=C bonds (Starodubtseva et al., 2008; Zhang et al., 2016). However, C_60_ is a hydrophobic nanomaterial; it has very low aqueous solubility and easily forms aggregates in water, which restricts its applicability (Jafvert and Kulkarni, 2008). Modification (such as carboxylation and hydroxylation) of the C_60_ surface enhances its water solubility and expands the range of possible applications (Mohan et al., 1998; Niu et al., 2011; Li et al., 2013; Lu et al., 2013; Hang et al., 2014; Lanzellotto et al., 2014; Cao et al., 2016; Xu et al., 2016; Najafi, 2017). Lanzellotto et al. (2014) constructed a Trametes versicolor laccase biosensor on Au-AuNC_60_OH. Fullerol enhanced the electron transfer between the active site of the enzyme and the electrode surface, leading to improved electrochemical biosensor performance. Tea polyphenols in beer were detected in the range of 0.03–0.30 mmol/L, with a limit of detection of 6 μmol/L.

Isocarbophos (IPS) is a fast-acting insecticide and acaricide that can cause poisoning by the esophagus, skin, and respiratory tract; all these have acute toxicity and cause cancer (Yamashita et al., 1997). The widespread uses of organic phosphorous insecticides indicate the extensive availability and potential for accidental and intentional human exposure (El-Behissy et al., 2001). Therefore, a rapid and accurate analytical method for the estimation of IPS is required. The main methods for IPS detection include chromatography (Huang et al., 2002; Yao et al., 2015; Li et al., 2017), chemiluminescence (Chen et al., 2012), and electrochemical methods (Yan et al., 2012). In recent years, new methods, such as highly selective aptamers and catalytic techniques, have been used to detect IPS (Pang et al., 2014; Zhang et al., 2014; Chen et al., 2017). In this paper, we propose nanocatalytic SPR spectroscopy for IPS detection, combining the favorable electron-transfer capabilities and catalytic behavior of fullerol to catalyze the sodium citrate–silver nitrate reaction and generate SPR on silver nanoparticles. The inhibitory effect on the catalytic reaction of the aptamer has been studied. Isocarbophos was selected as the target for a nanocatalytic SPR spectroscopy method. To the best of our knowledge, this is the first report describing the use of SPR (SERS, RRS, and Abs) combined with aptamer-labeled C_60_OH and AgNP (generated from the trisodium citrate–silver nitrate catalytic reaction) for the detection of IPS.

## Materials and Methods

### Apparatus

The following instrumentation was used: a DXR SmartRaman spectrometer (Thermo Company, USA) with a 633-nm laser at 3 mW power, a Cary Eclipse fluorescence spectrophotometer (Varian Company, USA), a TU-1901 double-beam UV-Visible spectrophotometer (Beijing General Instrument Co., LTD, China), and a FEI Quanta 200 FEG field-emission scanning electron microscope (Field Electron and Ion Company, Holland).

### Reagents

Aptamer (Apt) sequence of 5′-3′ AGC TTG CTG CAG CGA TTC TTG ATC GCC ACA GAG CT [Sangon Biotech (Shanghai) Co., Ltd., China], 0.01 mol/L silver nitrate (Sinopharm Chemical Reagent Co. Ltd., China), 0.2 g/L fullerene(C60), 0.04 g/L fullerol(C_60_OH), 0.1 g/L graphene oxide(GO) (Nanjing XFNANO Materials Tech Co., Ltd, China), 0.1 mol/L trisodium citrate (TSC) (Xilong Scientific Co., Ltd., China), 10.3 mol/L Victoria Blue B (VBB) solution, Victoria 4R (VB4R) solution, rhodamine S (RhS), rhodamine 6G (Rh6G, Sinopharm Chemical Reagent Co., Ltd., China), isocarbophos (Beijing Century OuKe Biological Technology Co., Ltd., China), profenofos (Sinopharm Chemical Reagent Co., Ltd., China), and glyphosate (J&K Scientific Ltd., China) were prepared. All reagents were analytically pure, and water was double-distilled.

To prepare fullerene (Andrievsky et al., 1995), 0.02 g fullerene was dissolved in 20 mL methylbenzene to give a bright purple solution. Double-distilled water (100 mL) was added, and the solution was sonicated until the toluene was completely volatilized. The solution changed to a dark-yellow suspension, and 0.2 g/L of fullerene sol was obtained.

Hydroxylated fullerene was prepared, referring to Li et al. (1998): 1 mL 0.2 g/L fullerene sol, 10 μL 30% H_2_O_2_ solution, and 100 μL 1 mol/L NaOH were mixed and reacted at room temperature. Then, 98 μL 1 mol/L hydrochloric acid was added to adjust pH to 7.5 and diluted 5 mL with water to obtain 0.04 g/L C_60_OH.

### Procedure

Apt (20 μL of a 1.5 μmol/L solution), a certain amount of IPS, and 10 μL of 0.04 g/L fullerol solution were added to a 5-mL graduated tube, mixed well, and allowed to react for 10 min. Next, 200 μL 0.01 mol/L AgNO_3_ and 70 μL 0.1 mol/L trisodium citrate were added and diluted to 1.5 mL. The mixture was heated for 21 min to 85°C in a water bath, then cooled with ice water. Next, 50 μL of 1.0 × 10^−5^ mol/L VBB and 40 μL of 1 mol/L NaCl were added and mixed well. SERS spectra were recorded using a Raman spectrometer. SERS intensity of the reaction solution at 1614 cm^−1^ ($I_{1614cm^{-1}}$) and the blank solution without IPS (*I*_0_) were recorded. The value of $\Delta I=I_{1614cm^{-1}}-I_{0}$ was calculated.

## Results and Discussion

### Principle

**Figure 1:**
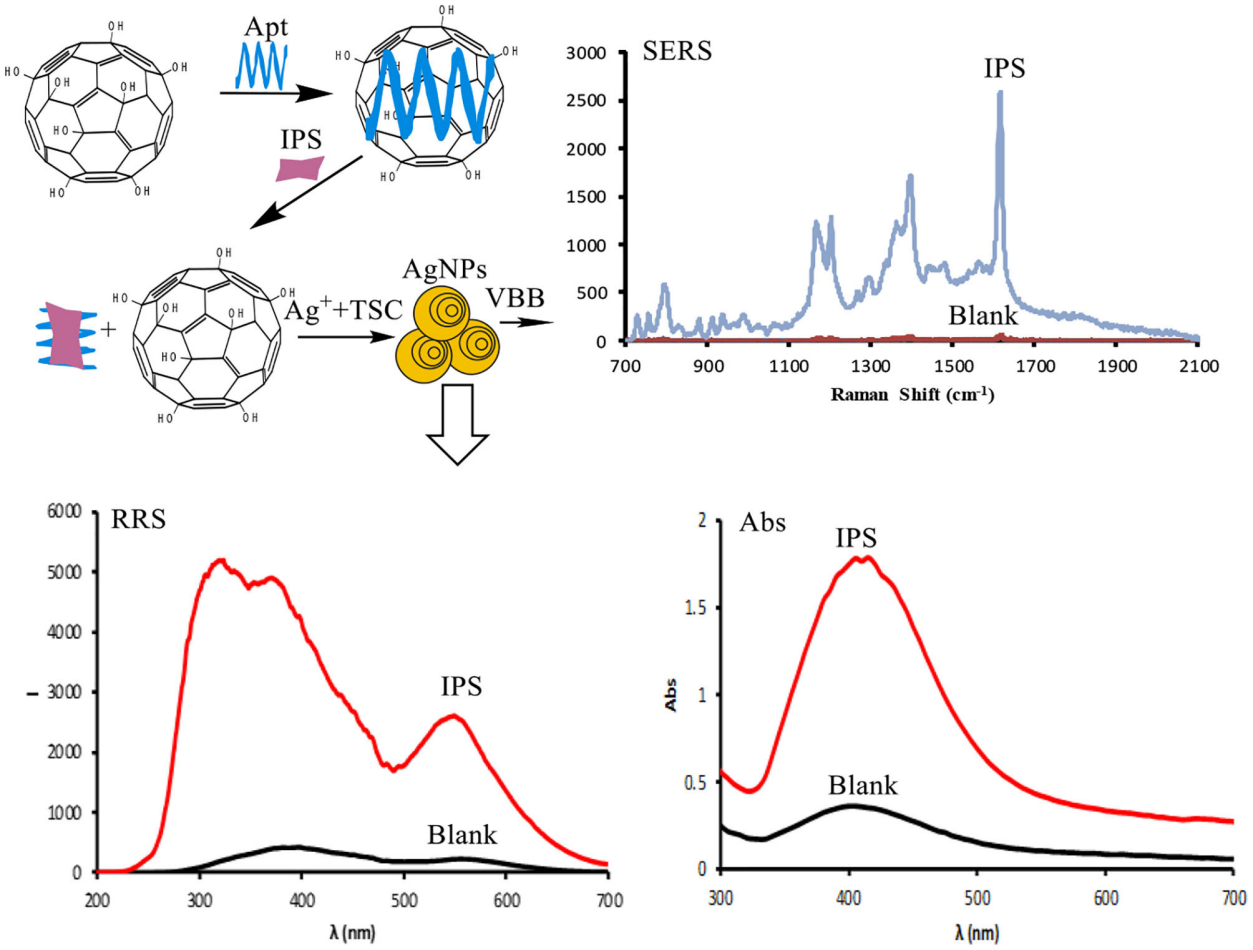
An aptamer trimode analytical platform for IPS based-C_60_(OH)_n_ catalytic amplification and AgNP trifunctional probex.

C_60_(OH)_n_ is a good electron acceptor (Samal and Sahoo, 1997). It transfers electrons from a donor to an acceptor, thereby facilitating, or catalyzing, the reaction. The silver nitrate–trisodium citrate reaction does not occur in solution because of the effective collision between silver ions and citrate. When C_60_(OH)_n_ is added, silver ions and citrate adsorb onto its surface, allowing electrons to transfer from citrate to silver ions, which leads to the generation of silver, 1,3-acetonedicarboxylic acid, and CO_2_. Silver assembles as yellow Ag nanoparticles (Figure 1). Fullerol has abundant hydroxyl groups that can form hydrogen bonds with water; it combines better with silver ions and citrate for more efficient electron transfer. Thus, fullerol has enhanced catalytic ability compared to fullerenes lacking the hydroxyl group. An aptamer coating on the fullerol surface hinders the interaction between C_60_(OH)_n_ and citrate and silver ions, such that C_60_(OH)_n_ catalytic activity is inhibited. In the presence of IPS specific to the aptamer, C_60_(OH)_n_ is once again exposed to the reaction system and its catalytic activity is recovered. The amount of Ag nanoparticles generated increases linearly with IPS concentration. Using this relationship, a method to detect IPS using the SPR absorption spectrum, RRS, and SERS was developed.

### SERS Spectra

**Figure 2:**
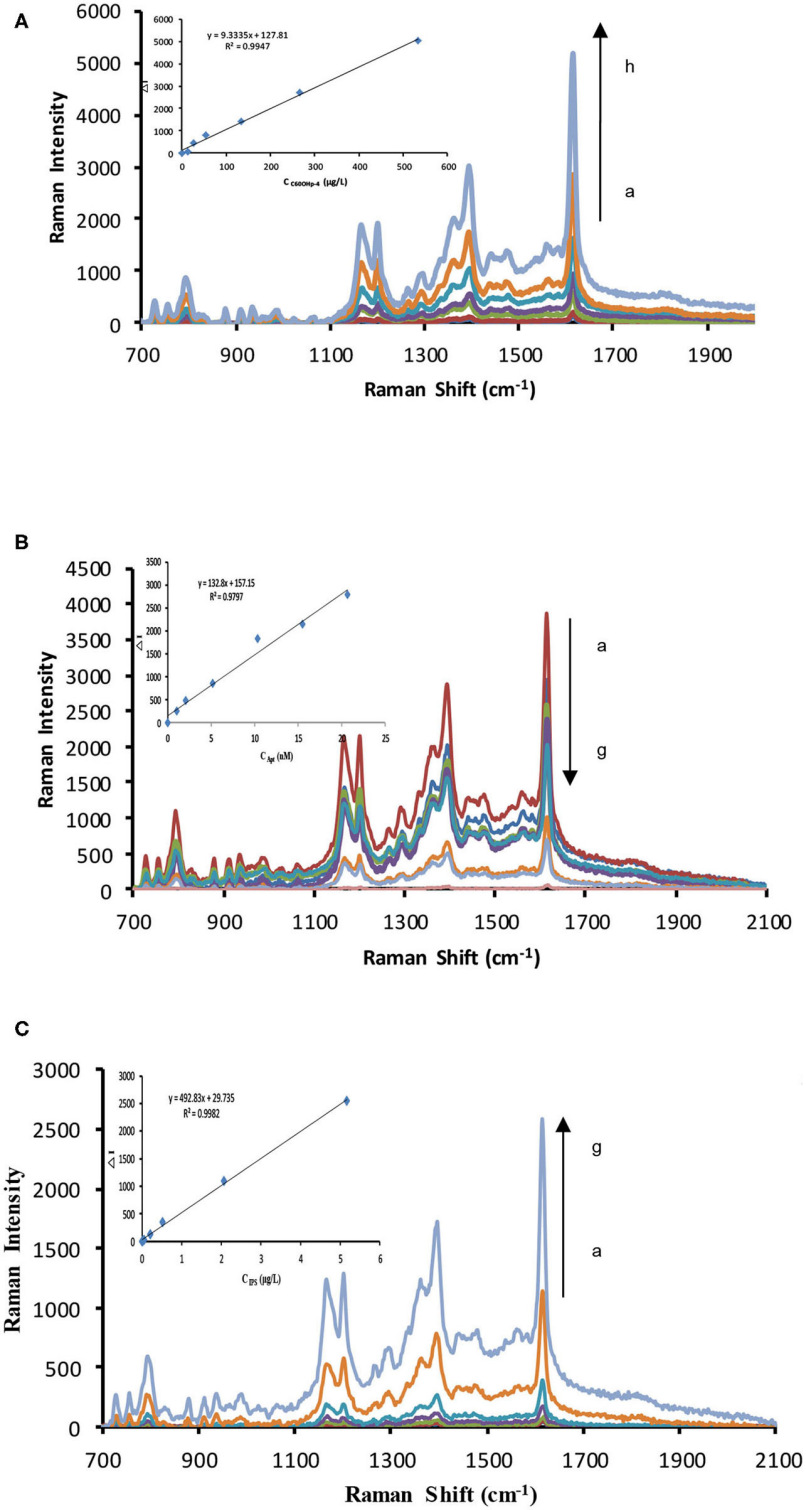
SERS spectra. **(A)** C_60_OH–AgNO_3_-TSC catalytic system. (0, 2.67, 5.33, 13.33, 26.67, 53.33, 133.33, 266.67, 533.33 μg/L) C_60_OH_4_+ 1.33 mmol/L AgNO_3_ + 4.67 mmol/L TSC +85°C+21 min +3.33×10^−7^ mol/L VBB+0.02 mol/L NaCl. **(B)** Apt–C_60_OH–AgNO_3_-TSC inhibitory catalytic system. (0, 1.03, 2.07, 5.17, 10.33, 15.5, 20.67 nmol/L) Apt + 266.67 μg/L C_60_OH+21 min +1.33 mmol/L AgNO_3_+4.67 mmol/L TSC+85°C+3.33×10^−7^ mol/L VBB+0.02 mol/L NaCl. **(C)** Apt–C_60_OH–AgNO_3_-TSC-IPS detection system. 20.67 nmol/L Apt + 266.67 μg/L C_60_OH+ (0, 0.02, 0.05, 0.21, 0.52, 2.07, 5.17 μg/L) IPS + 1.33 mmol/L AgNO_3_+ 4.67 mmol/L TSC+85°C+21 min +3.33×10^−7^ mol/L VBB+0.02 mol/L NaCl.

In this study, fullerol exhibited increased catalytic activity compared to C_60_. Fullerol has –OH groups with excellent water solubility that increase its catalytic activity compared to C_60_. Thus, fullerols were prepared by H_2_O_2_ oxidization using a previously published procedure (Li et al., 1998). Hydroxyl content in fullerol increases with increasing H_2_O_2_ (Figure S1), as does the catalytic action of silver nitrate–trisodium citrate; a 70-mmol/L H_2_O_2_ solution was selected to obtain highly catalytic fullerol (C_60_OH). VBB, VB4R, RhS, Rh6G, and RhB were used as signal molecules; their strongest SERS peaks occurred at 1614, 1385, 1361, 1362, and 1508 cm^−1^, respectively (Figure S2). The SERS intensities of VBB and VB4R were stronger than those of the others; VBB was chosen for further study. The catalytic activities of C_60_OH, C_60_, GO, and AgNP were investigated (Figure 2A and Figure S3). C_60_OH exhibited the highest catalytic activity and was chosen for use. In the presence of Apt, which coats the fullerol surface and isolates it from the reactants, fullerol catalytic activity is suppressed and decreased SERS intensity is observed (Figure 2B). When added to the system, IPS conjugates to Apt, releasing fullerol and restoring its catalytic activity. As the IPS concentration increases, the amount of released fullerol increases and more AgNP is produced as well; thus, SERS intensity increases linearly with IPS concentration (Figure 2C and Figure S4).

### RRS and Absorption Spectra

**Figure 3:**
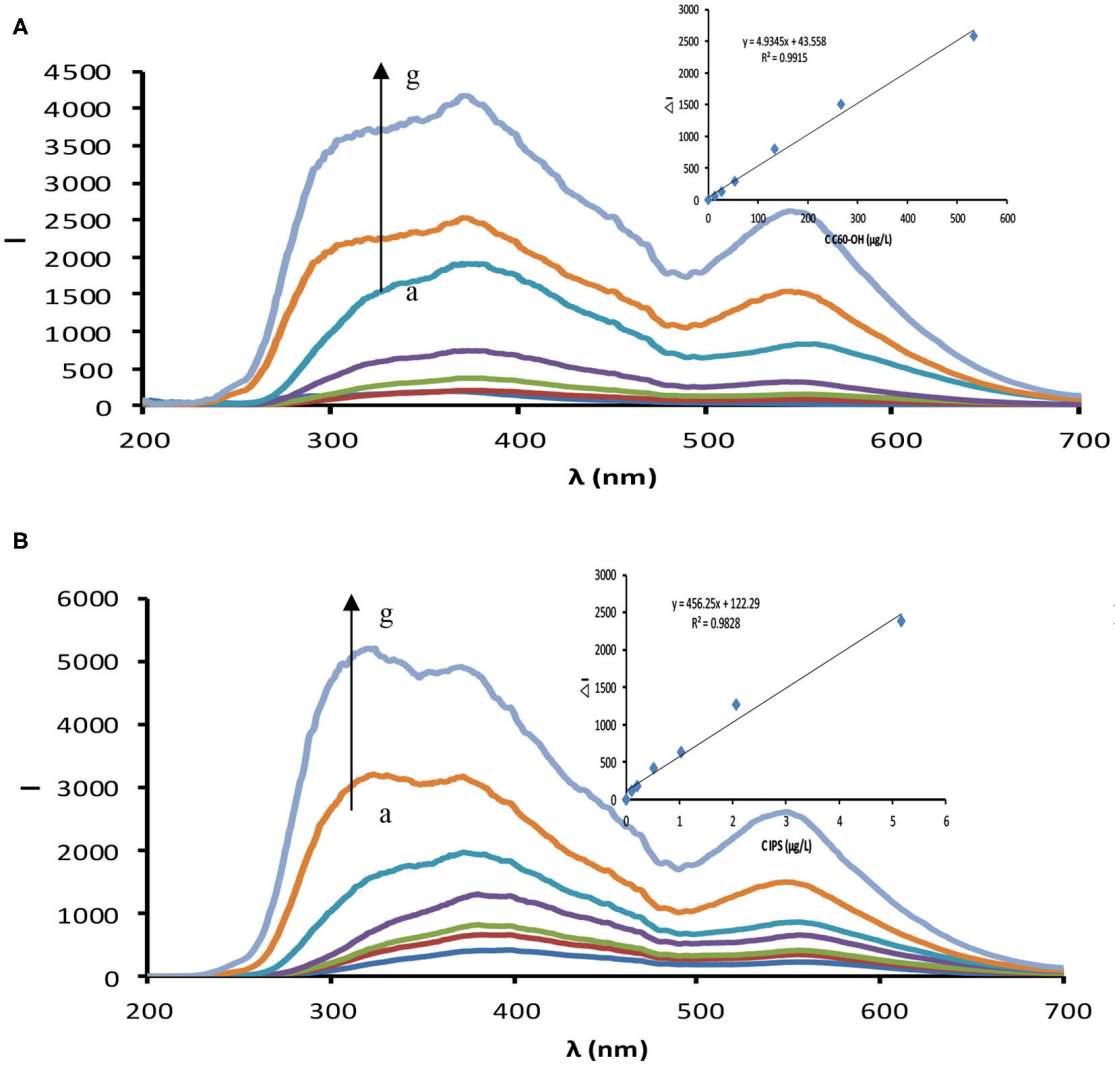
RRS spectra. **(A)** C_60_OH–AgNO_3_-TSC catalytic system, (0, 13.33, 26.67, 53.33, 133.33, 266.67, 533.33 μg/L) C_60_OH+ 1.33 mmol/L AgNO_3_+ 4.67 mmol/L TSC +85°C+21 min +3.33×10^−7^ mol/L VBB+0.02 mol/L NaCl. **(B)** Apt–C_60_OH–AgNO_3_-TSC-IPS detection system, 20.67 nmol/L Apt + 266.67 μg/L C_60_OH + (0, 0.1, 0.21, 0.52, 1.03, 2.07, 5.17 μg/L) IPS + 1.33 mmol/L AgNO_3_+ 4.67 mmol/L TSC +85°C+21 min +3.33×10^−7^ mol/L VBB+0.02 mol/L NaCl.

**Figure 4:**
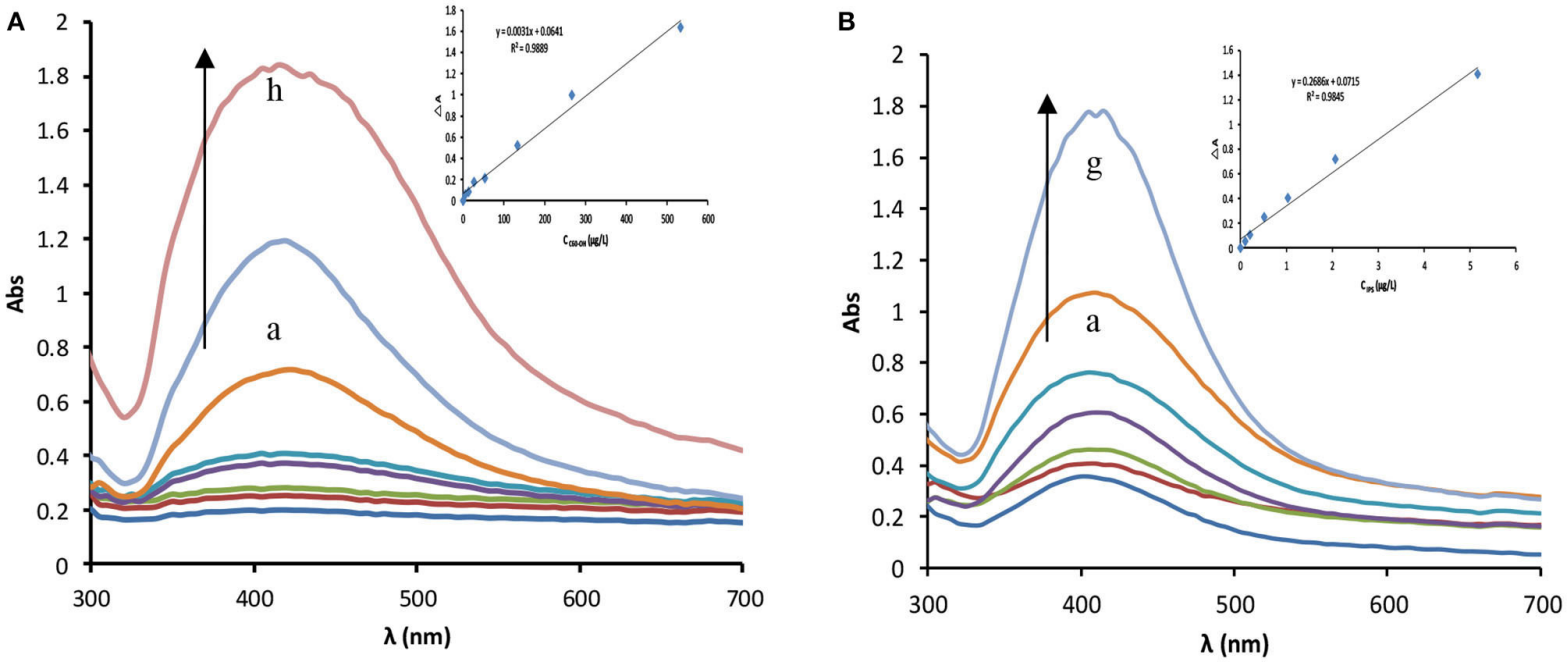
Absorption spectra. **(A)** C_60_OH–AgNO_3_-TSC catalytic system, (0, 5.33, 13.33, 26.67, 53.33, 133.33, 266.67, 533.33 μg/L) C_60_OH + 1.33 mmol/L AgNO_3_ + 4.67 mmol/L TSC +85°C+21 min +3.33×10^−7^ mol/L VBB+0.02 mol/L NaCl. **(B)** Apt–C_60_OH–AgNO_3_-TSC-IPS detection system, 20.67 nmol/L Apt + 266.67 μg/L C_60_OH + (0, 0.1, 0.21, 0.52, 1.03, 2.07, 5.17 μg/L) IPS + 1.33 mmol/L AgNO_3_+ 4.67 mmol/L TSC +85°C+21 min +3.33×10^−7^ mol/L VBB+0.02 mol/L NaCl.

In a water bath at 85°C, fullerol and other nanoparticles catalyze the reaction of silver nitrate and trisodium citrate to generate AgNP, which exhibits two strong RRS peaks at 360 and 550 nm (Figure 3 and Figure S5A) and a strong surface plasma resonance (SPR) absorption peak at 410 nm (Figure 4 and Figure S6A). The RRS peak at 550 nm is characteristic of AgNP, and the intensity of Δ*I*_550nm_ and Δ*A*_410nm_ increase linearly with the amount of nanocatalyst. When Apt coats the nanocatalyst surface, it isolates the nanocatalyst from the system and inhibits its catalytic activity, leading to decreased Δ*I*_550nm_ and Δ*A*_410nm_ values. In the presence of IPS, which conjugates specifically with Apt, fullerol is released and its catalytic activity recovers. As IPS concentration increases, the amount of released fullerol increases, as does the amount of AgNP generated; consequently, Δ*I*_550nm_ and Δ*A*_410nm_ intensities increase linearly with IPS concentration (Figures 3B, 4B and Figures S5B, S6B).

### The Catalytic Effect of C_60_OH and Inhibition of the Aptamer

In the absence of the catalyst, AgNO_3_ does not react readily with trisodium citrate. However, in the presence of a fullerol nanocatalyst, silver ions and citrate adsorb to the fullerol surface by interface free energy. As shown in Figure S7, the intensity of RRS for fullerol in aqueous solution is considerably lower than that of fullerene, suggesting that the fullerol particle size is less than that of fullerene. This may be responsible for the reduced catalytic activity of fullerenes compared to fullerol. In addition, silver ions and citrate adsorb to the surface more readily and electron transfer between the silver and citrate ions occurs more efficiently. That is, smaller particles demonstrate greater catalytic efficiency. As shown in Table 1, the slope of the C_60_OH catalytic system is about 50 times that of C_60_. In addition, the catalytic effect of AgNP on this reaction was studied. As shown in Figure S8 and Table 1, AgNP is an effective catalyst even with AgNP concentrations as low as 13.33 nmol/L. AgNP participates in autocatalysis, strengthening the catalytic effect. Furthermore, fullerene was hydroxylated using H_2_O_2_, according to a previously published procedure [42] and its catalytic activity was determined. Fullerol exhibited enhanced catalytic activity (compared to fullerene), and its catalytic activity increased with increasing hydroxylation (Table 1). This suggests that improved solubility of fullerol in water would increase its ability to bind with ions, thereby enhancing catalysis. When Apt is added, the intensity of RRS increases, as shown in Figure S7C, indicating that Apt coats the nanocatalyst surface and blocks the adsorption of silver and citrate ions to the nanocatalyst, inhibiting its catalytic activity. It is worth mentioning that the catalytic activity of fullerol is suppressed by Apt (Table 1). This is likely due to its smaller size; the hydroxyl group of fullerol produced a better combination of hydroxyl and –COOH, –NH_2_.

**Table 1:** The catalytic effect of various catalyst and the inhibiting effect of Apt.

**System**	**Linear equation**	**Linearity range**	**Correlation**
			**coefficient (*R*^**2**^)**
C_60_	*Δ*$I_{1614cm^{-1}}$ = 0.24 *C* + 155.77	133.33–13333.33 μg/L	0.9954
C_60_OH	*Δ$I_{1614cm^{-1}}$* =9.33 *C* + 127.81	13.33–533.33 μg/L	0.9947
C_60_OH_P_	*Δ$I_{1614cm{-}^{1}}$* =9.33 *C* + 127.81	13.33–533.33 μg/L	0.9947
AgNP	*Δ$I_{1614cm^{-1}}$* = 11.36 *C* + 50.21	1.44~359.56 μg/L	0.9979
Apt-C_60_OH	*Δ$I_{1614cm{-}^{1}}$* = 132.8 *C* + 157.15	1.03~20.67 nmol/L	0.9797
Apt-C_60_	*Δ$I_{1614cm^{-1}}$* = 21.41 *C* – 9.85	5.17~51.67 nmol/L	0.9913

### Scanning Electron Microscopy (SEM)

**Figure 5:**
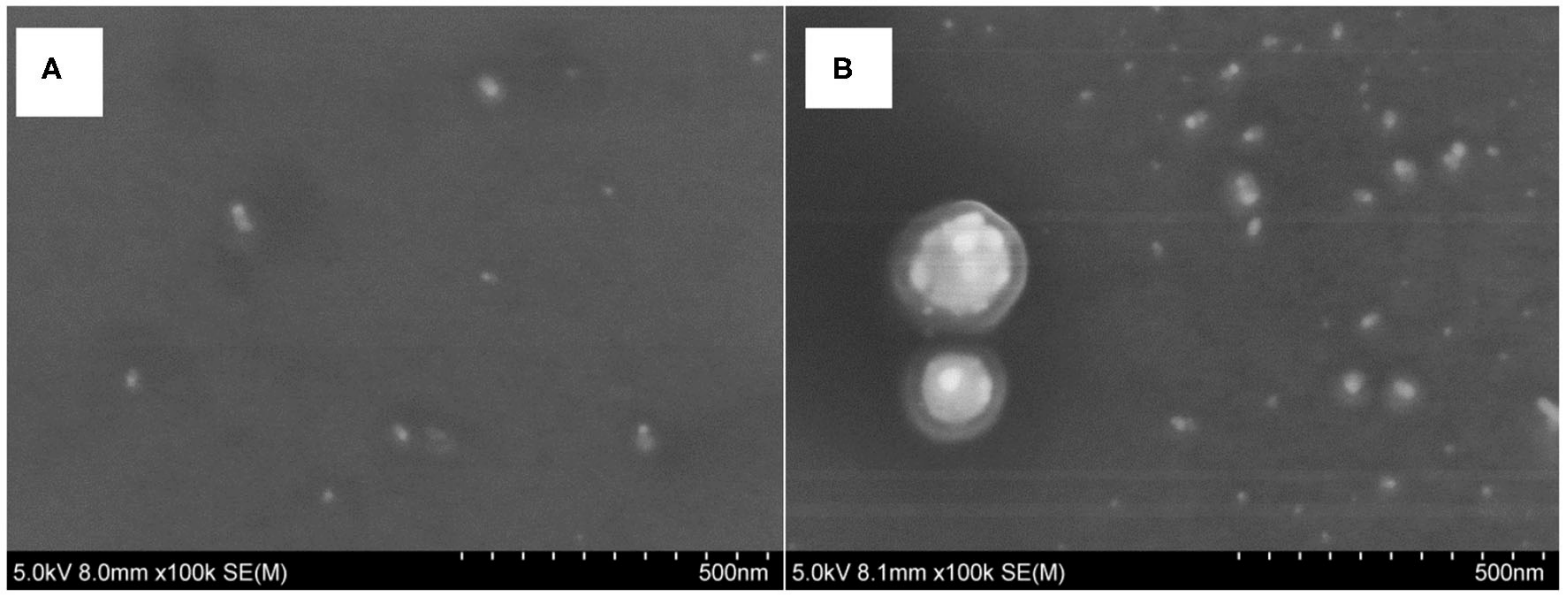
SEM of aptamer–C_60_OH–AgNO_3_-TSC-IPS detection system. **(A)** 20.67 nmol/L Apt + 266.67 μg/L C_60_OH + 1.33 mmol/L AgNO_3_+ 4.67 mmol/L TSC +85°C+21 min; **(B)** a+ 0.21 μg/L IPS.

The reaction solution was prepared and diluted 10 times. A 10 μL sample solution was dropped onto a silicon wafer and allowed to dry naturally, then scanning electron microscopy (SEM) was performed. As shown in Figure 5, in the absence of IPS, few AgNPs are detected in the reaction solution, with a mean grain size of 20 nm (Figure 5A). Upon addition of IPS, the catalyst recovered catalytic activity; a large amount of AgNP was generated and formed aggregates with a mean grain size of 40 nm (Figure 5B).

### Optimization of Catalysis Conditions

The effect of reagent concentration on the determination was studied. When AgNO_3_ and TSC concentrations were 1.33 and 4.67 mmol/L, respectively, the SERS value was at its maximum. Thus, 1.33 mmol/L AgNO_3_ and 4.67 mmol/L TSC were chosen as the optimal concentrations (Figures S9, S10). The effects of reaction temperature and time were tested as well; 85°C and 21 min resulted in the maximum value for ΔI (Figures S11, S12). The effects of VBB, VB4R, RhB, RhS, and Rh6G concentrations on ΔI were considered. Maximum ΔI was observed at VBB, VB4R, RhB, RhS, and Rh6G concentrations of 3.33 × 10^−7^ mol/L, 1 × 10^−6^ mol/L, 1 × 10^−5^ mol/L, 1.67 × 10^−6^ mol/L, and 1 × 10^−6^ mol/L, respectively. Among these, VBB was selected because of its lower detection limit (Figure S13). At an Apt concentration of 20.67 nmol/L, the ΔI value reached a maximum value and thus was chosen for use (Figure S14). Binding times for the aptamers with fullerol were tested. A maximum value of ΔI was reached and maintained at 8 min; thus, it was chosen as the optimal binding time (Figure S15).

### Working Curve

**Figure 6:**
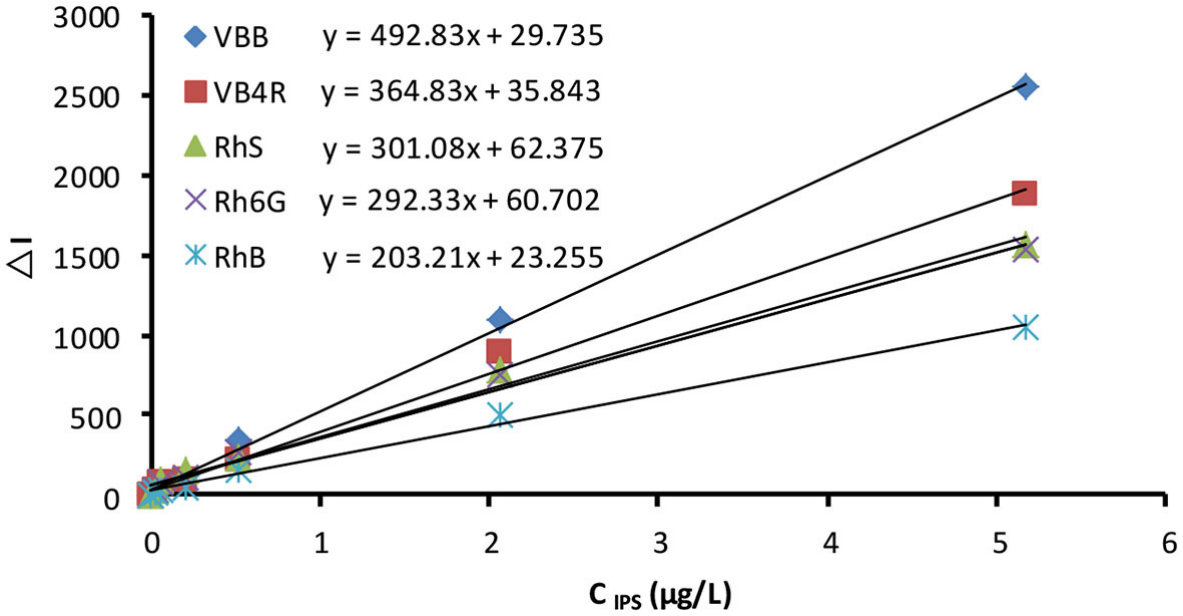
Working curve for the SERS determination of Apt–C_60_OH–AgNO_3_-TSC-IPS. 20.67 nmol/L Apt + 0.05–5 μg /L IPS + 266.67 μg/L C_60_OH + 1.33 mmol/L AgNO_3_+ 4.67 mmol/L TSC+85°C+ 21 min +3.33×10^−7^ mol/L VBB+0.02 mol/L NaCl.

Using the optimal conditions described in section Optimization of Catalysis Conditions, working curves were prepared for IPS concentration at the corresponding Δ$I_{1614cm^{-1}}$, Δ*I*_550nm_, and Δ*A*_410nm_ values for SERS, RRS, and Abs, respectively (Figure 6 and Figures S16–S19). Analytical characteristics are listed in Table 2. SERS exhibited the best performance, with a maximum slope of 492.83, and a limit of detection of 8.2 ng/L; RRS was the next most effective method. However, the Abs method is inexpensive, convenient, and aligns with national standards such that it could be used for on-site tests. Fullerol, because of its small size, higher surface electronic density, and ability to bind with silver and citrate ions, displays enhanced catalytic activity and a greater sensitivity for IPS detection compared to fullerene. Organophosphate analogs glyphosate, profenofos, and tributylphosphine also were detected using this method, according to the linear equations $I_{1614cm^{-1}}=0.93C_{IPS}+93.71$, $I_{1614cm^{-1}}=0.31C_{IPS}+75.53$, and $I_{1614cm^{-1}}=0.87C_{IPS}+66.21$, respectively (Figure S20). These detection ranges exceeded that of IPS, and the components did not interfere with the determination. Compared to previously reported methods for the determination of IPS, the SERS, RRS, and Abs method (Table S1) is simpler, requires an easily obtainable reagent, is highly sensitive, and exhibits good selectivity. It can be used to detect IPS residues in water and agricultural products.

**Table 2:** Analytical characteristics of the aptamer adjust catalysis-Ag nano plasma SERS for the determination of IPS.

**Test method**	**System**	**Working curve**	**Linearly range**	**Limit of detection**	**Coefficient (*R*^**2**^)**
SERS	C_60_OH-VBB	*Δ*$I_{1614cm^{-1}}$** = 492.83 *C* + 29.74	0.02~5.17 μg/L	8.2 ng/L	0.9982
	C_60_OH-VB4R	*Δ$I_{1385cm^{-1}}$* = 364.83 *C* + 35.84	0.02~5.17 μg/L	9.1 ng/L	0.9945
	C_60_OH-RhS	*Δ$I_{1361cm^{-1}}$* = 301.08 *C* + 62.38	0.02~5.17 μg/L	8.7 ng/L	0.9904
	C_60_OH-Rh6G	*Δ$I_{1362cm^{-1}}$* = 292.33 *C* + 60.7	0.02~5.17 μg/L	10.3 ng/L	0.9906
	C_60_OH-RhB	*Δ$I_{1508cm^{-1}}$* = 203.21 *C* + 23.26	0.02~5.17 μg/L	10.1 ng/L	0.9946
	C_60_-VBB	*Δ$I_{1614cm^{-1}}$* = 78.41 *C* + 44.20	0.21~15.5 μg/L	0.03 μg/L	0.9954
	GO-VBB	*Δ$I_{1614cm^{-1}}$* = 80.67 *C* + 64.34	0.52~15.5 μg/L	0.2 μg/L	0.9816
	AgNVBB	*Δ$I_{1614cm^{-1}}$* = 361.28 *C* + 69.57	0.02~5.17 μg/L	10.2 ng/L	0.9824
RRS	C_60_OH	*ΔI_*550*nm**_* = 456.26 *C* + 122.29	0.1~5.17 μg/L	0.02 μg/L	0.9828
	C_60_	*ΔI_*550*nm**_* = 183.95 *C* + 46.36	0.1~5.17 μg/L	0.02 μg/L	0.9883
Abs	C_60_OH	*ΔA_*410*nm**_* = 0.27 *C* + 0.07	0.52~15.5 μg/L	0.03 μg/L	0.9845
	C_60_	*ΔA_*410*nm**_* = 0.05 *C* – 0.0014	0.52~15.5 μg/L	0.04 μg/L	0.9917

### Influence of Substances

Using fullerol as a catalyst, the influence of coexisting substances on the determination of 2 μg/L IPS was tested. The results indicate that common substances do not interfere with IPS determination (Table S2), with a relative error of ±10%.

### Sample Analysis

Three water samples, taken from a pond, Lijiang, and cropland were collected using two 100 mL glass sampling bottles and were then filtered through a 150 nm filter membrane to obtain sample solutions, which were stored at 4°C. Food samples (200 g grape, 265 g orange (3), and 200 g Chinese cabbage) were purchased from farmer markets. The samples were immersed in 100 mL of acetone for 2 h. Extracts were air-dried, then dissolved with sonication in 100 mL water, and then stored at 4°C. Samples (50 μL) were then tested for IPS content. A known amount of IPS was added to each sample, and recoveries of 93–101.5% were obtained (Table 3).

**Table 3:** Sample analysis results (*n* = 5).

**Sample**	**Found**	**Added**	**Found**	**Recovery/%**
Pond water	–	2 μg/L	2.01 μg/L	100.5%
Lijiang River	–	2 μg/L	1.95 μg/L	97.5%
Cropland 1	–	2 μg/L	2.03 μg/L	101.5%
Cropland 2	0.22 μg/L	2 μg/L	2.28 μg/L	103%
Grape	–	2 μg/L	1.86 μg/L	93%
Orange	–	2 μg/L	1.92 μg/L	96%
Chinese cabbage	–	2 μg/L	2.01 μg/L	100.5%
Pond water	–	2 μg/L	2.01 μg/L	100.5%

## Conclusions

C_60_OH is an effective catalyst to generate yellow AgNP via the AgNO_3_-trisodium citrate reaction. The generated AgNPs exhibit a strong plasma resonance effect that increases linearly with catalyst amount at a certain concentration. RRS spectra demonstrate that the abundant hydroxyl groups of C_60_OH increase its hydrophilicity and its ability to bind silver and citrate ions, resulting in increased catalytic activity compared to C_60_. When C_60_OH is coated with an aptamer, silver ions cannot bind to C_60_OH, and catalytic activity is suppressed. Conversely, when isocarbophos conjugates with the specific aptamer, C_60_OH is released and catalytic activity is recovered. SPR (Abs, RRS, and SERS) intensities increased linearly with increasing IPS concentration. Thus, aptamer binding and nanocatalysis combine with SPR to provide a sensitive, selective, simple, and rapid method for the determination of IPS.

## Data Availability Statement

All datasets presented in this study are included in the article/Supplementary Material.

## Author Contributions

AL and ZJ conceived and designed the experiments. HO performed the experiments, analyzed the data, and wrote the paper. All authors contributed to the article and approved the submitted version.

## Conflict of Interest

The authors declare that the research was conducted in the absence of any commercial or financial relationships that could be construed as a potential conflict of interest.

## References

[B1] Alvarez-Puebla R. A. Liz-Marzan L. M. (2012). SERS detection of small inorganic molecules and ions. Angew. Chem. Int. Ed. 51, 11214–11223. 10.1002/anie.201204438 23074161

[B2] Andrievsky G. V. Kosevich M. V. Vovk O. M. Shelkovsky V. S. Vashchenko L. A. (1995). On the production of an aqueous colloidal solution of fullerenes. J. Chem. Soc. Chem. Commun. 98, 1281–1282. 10.1039/c39950001281

[B3] Cai Q. Hu Z. F. Zhang Q. Li B. Y. Shen Z. R. (2017). Fullerene (C_60_)/CdS nanocomposite with enhanced photocatalytic activity and stability. Appl. Surf. Sci. 403, 151–158. 10.1016/j.apsusc.2017.01.135

[B4] Cao T. T. Wang Z. W. Xia Y. J. Song B. Zhou Y. Chen N.. (2016). Facilitating electron transportation in perovskite solar cells via water-soluble fullerenol interlayers. ACS Appl. Mat. Inter. 8, 18284–18291. 10.1021/acsami.6b04895 27311625

[B5] Chen D. Song Z. Lv H. (2012). Assay of picogram level isocarbophos residue on tangerines and oranges with luminol-albumin chemiluminescence system. Food Chem. 135, 2549–2553. 10.1016/j.foodchem.2012.07.014 22980841

[B6] Chen H. Y. Wu Y. G. Yang W. P. Zhan S. S. Qiu S. Y. Zhou P. (2017). Ultrasensitive and selective detection of isocarbophos pesticide basedon target and random ssDNA triggered aggregation of hemin in polarorganic solutions. Sensor. Actuat. B Chem. 243, 445–453. 10.1016/j.snb.2016.12.014

[B7] El-Behissy E. Y. King R. D. Ahmed M. M. Youssef A. M. (2001). Fate of postharvest-applied dichlorvos in stored and processed dates. Agric. J. Food Chem. 49, 1239–1245. 10.1021/jf000812e 11312843

[B8] Gao L. Z. Zhuang J. Nie L. Zhang J. B. Zhang Y. Gu N.. (2007). Intrinsic peroxidase-like activity of ferromagnetic nanoparticles. Nat. Nanotechnol. 9, 577–583. 10.1038/nnano.2007.260 18654371

[B9] Hang L. Wang Q. X. Gao F. Shi J. L. Gao F. (2014). A high-performance DNA biosensor using polyhydroxylated fullerenol as 3D matrix for probe immobilization. Electrochem. Commun. 47, 84–87. 10.1016/j.elecom.2014.07.025

[B10] Huang G. M. Ouyang J. Baeyens W. R. G. Yang Y. P. Tao C. J. (2002). High-performance liquid chromatographic assay of dichlorvos, isocarbophos and methyl parathion from plant leaves using chemiluminescence detection. Anal. Chim. Acta. 474, 21–29. 10.1016/S0003-2670(02)01014-0

[B11] Jackman J. A. Rahim F. A. Cho N. J. (2017). Nanoplasmonic sensors for biointerfacial science. Chem. Soc. Rev. 46, 3615–3660. 10.1039/C6CS00494F 28383083

[B12] Jafvert C. T. Kulkarni P. P. (2008). Buckminsterfullerene's (C60) octanol-water partition coefficient (Kow) and aqueous solubility. Environ. Sci. Technol. 42, 5945–5950. 10.1021/es702809a 18767649

[B13] Jiang Z. L. Fan Y. Y. Liang A. H. Wen G. Q. Liu Q. Y. Li T. S. (2010a). Resonance scattering spectral detection of trace Pb^2+^ using aptamer-modified AuPd nanoalloy as probe. Plasmonics. 5, 375–381. 10.1007/s11468-010-9153-8

[B14] Jiang Z. L. Liao X. J. Deng A. P. Liang A. H. Li J. S. Pan H. C.. (2008). Catalytic effect of nanogold on Cu(II)-N_2_H_4_ reaction and its application to resonance scattering immunoassay. Anal. Chem. 80, 8681–8687. 10.1021/ac801647b 18928303

[B15] Jiang Z. L. Zhang J. Wen G. Q. Liang A. H. Liu Q. Y. Kang C. Y. (2010b). Aptamer-modified AuRe nanoalloy probe for trace Hg^2+^ using resonance scattering as detection technique. Chinese J. Chem. 28, 1159–1164. 10.1002/cjoc.201090201

[B16] Justino C. I. L. Gomes A. R. Freitas A. C. Duarte A. C. Rocha-Santos T. A. P. (2017). Graphene based sensors and biosensors. Trend Anal. Chem. 91, 53–66. 10.1016/j.trac.2017.04.003

[B17] Kotov N. A. (2010). Chemistry. Inorganic nanoparticles as protein mimics. Science 330, 188–189. 10.1126/science.1190094 20929766

[B18] Krätschmer W. Lamb L. D. Fostiropoulos K. Huffman D. R. (1990). Solid C60: a new form of carbon. Nature 347, 354–358. 10.1038/347354a0

[B19] Lanzellotto C. Favero G. Antonelli M. L. Tortolini C. Cannistraro S. Coppari E.. (2014). Nanostructured enzymatic biosensor based on fullerene and gold nanoparticles: preparation, characterization and analytical applications. Biosens. Bioelectron. 55, 430–437. 10.1016/j.bios.2013.12.028 24441023

[B20] Li D. Q. Jiang M. D. Xu L. H. Qiao X. G. Xu Z. X. (2017). Simultaneous determination of acephate and isocarbophos in vegetables by capillary electrophoresis using ionic liquid and sodium dodecyl sulfate as modifiers. Food Anal. Method. 10, 3368–3374. 10.1007/s12161-017-0897-z

[B21] Li R. M. Zhen M. M. Guan M. R. Chen D. Q. Zhang G. Q. Ge J. C.. (2013). A novel glucose colorimetric sensor based on intrinsic peroxidase-like activity of C60-carboxyfullerenes. Biosens. Bioelectron. 47, 502–507. 10.1016/j.bios.2013.03.057 23628844

[B22] Li T. B. Huang K. X. Li X. H. Jiang H. Y. Li J. Yan X. Z. (1998). Studies on the rapid preparation of fullerols and its formation mechanism. Chem. J. Chinese U. 19, 858–860.

[B23] Liang A. H. Shang G. Y. Ye L. L. Wen G. Q. Luo Y. H. Liu Q. Y. (2015). A SERS nanocatalytic reaction and its application to quantitative analysis of trace Hg(II) with Vitoria blue B molecular probe. Rsc Adv. 5, 21326–21331. 10.1039/C4RA16110F

[B24] Liang A. H. Zhang Y. Fan Y. Y. Chen C. Q. Wen G. Q. Liu Q. Y.. (2011). Catalysis of aptamer-modified AuPd nanoalloy probe and its application to resonance scattering detection of trace ${\text{UO}}_{2}^{2+}$. Nanoscale 3, 3178–3184. 10.1039/c1nr10275c 21677977

[B25] Lu X. Q. Shan D. L. Yang J. M. Huang B. M. Zhou X. B. (2013). Determination of m-dinitrobenzene based on novel type of sensor using thiol-porphyrin mixed monolayer-tethered polyaniline with intercalating fullerenols. Talanta 115, 457.461 10.1016/j.talanta.2013.06.002 24054618

[B26] Mohan H. Palit D. K. Mittal J. P. Chiang L. Y. Asmusc K. D. Guldi D. M. (1998). Excited states and electron transfer reactions of C60(OH)18 in aqueous solution. J. Chem. Soc. Faraday Trans. 94, 359–363. 10.1039/a705293f

[B27] Najafi A. (2017). An investigation on dispersion and stability of water-soluble fullerenol (C_60_OH) in water via UV–Visible spectroscopy. Chem. Phys. Lett. 669, 115–118. 10.1016/j.cplett.2016.12.030

[B28] Niu F. Wu J. Y. Zhang L. S. Li P. Zhu J. F. Wu Z. Y. (2011). Hydroxyl group rich C_60_ fullerenol: an excellent hydrogen bond catalyst with superb activity, selectivity, and stability. ACS Catal. 1, 1158–1161. 10.1021/cs200317d

[B29] Ouyang H. X. Li C. N. Liu Q. Y. Wen G. Q. Liang A. H. Jiang Z. L. (2017). Resonance Rayleigh scattering and SERS spectral detection of trace Hg(II) based on the gold nanocatalysis. Nanomaterials 7:114. 10.3390/nano7050114 28513536 PMC5449995

[B30] Pang S. T. Labuza T. P. He L. L. (2014). Development of a single aptamer-based surface enhanced Raman scattering method for rapid detection of multiple pesticides. Analyst 139, 1895–1901. 10.1039/C3AN02263C 24551875

[B31] Samal S. Sahoo S. K. (1997). An overview of fullerene chemistry. Bull. Mater. Sci. 20, 141–230. 10.1007/BF02744892

[B32] Starodubtseva E. V. Sokolov V. I. Bashilov V. V. Novikov Y. N. Martynova E. V. Vinogradov M. G. (2008). Fullerene complexes with palladium and rhodium as catalysts for acetylenic bond hydrogenation. Mendeleev. Commun. 18, 209–210. 10.1016/j.mencom.2008.07.014

[B33] Wei H. Wang E. K. (2013). Nanomaterials with enzyme-like characteristics (nanozymes): next-generation artificial enzymes. Chem. Soc. Rev. 42, 6060–6093. 10.1039/c3cs35486e 23740388

[B34] Xu T. Y. Zhu R. L. Liu J. Zhou Q. Zhu J. X. Liang X. L. (2016). Fullerol modification ferrihydrite for the degradation of acid red 18under simulated sunlight irradiation. J. Mol. Catal. A Chem. 424, 393–401. 10.1016/j.molcata.2016.09.024

[B35] Yamashita M. Tanaka J. Ando Y. (1997). Human mortality in organophosphate poisoning. Toxicol. App. 39, 84–85. 9080632

[B36] Yan X. N. Deng J. Xu J. S. Li H. Wang L. L. Chen D. (2012). A novel electrochemical sensor for isocarbophos based on a glassy carbon electrode modified with electropolymerized molecularly imprinted terpolymer. Sensor. Actuat. B Chem. 171–172, 1087–1094. 10.1016/j.snb.2012.06.038

[B37] Yao D. M. Wen G. Q. Jiang Z. L. (2013). A highly sensitive and selective resonance Rayleigh scattering method for bisphenol A detection based on the aptamer–nanogold catalysis of the HAuCl_4_-vitamin C particle reaction. Rsc. Adv. 3, 13353–13356. 10.1039/c3ra41845f

[B38] Yao Z. L. Lin M. Xu M. F. Wang T. Y. Ping X. L. Wu S. H. (2015). Simultaneous enantioselective determination of isocarbophos and its main metabolite isocarbophos oxon in rice, soil, and water by chiral liquid chromatographyand tandem mass spectrometry. J. Sep. Sci. 38, 1663–1672. 10.1002/jssc.201500155 25755196

[B39] Ye L. L. Wen G. Q. Ouyang H. X. Liu Q. Y. Liang A. H. Jiang Z. L. (2016). A novel and highly sensitive nanocatalytic surface plasmon resonance-scattering analytical platform for detection of trace Pb ions. Sci. Rep. 6:24150. 10.1038/srep24150 27071936 PMC4829859

[B40] Zhang C. Z. Wang L. Tu Z. Sun X. He Q. H. Lei Z. J.. (2014). Organophosphorus pesticides detection using broad-specific single-stranded DNA based fluorescence polarization aptamer assay. Biosens. Bioelectron. 55, 216–219. 10.1016/j.bios.2013.12.020 24384262

[B41] Zhang L. Holt C. M. B. Luber E. J. Olsen B. C. Wang H. T. Danaie M. (2011). High rate electrochemical capacitors from three-dimensional arrays of vanadium nitride functionalized carbon nanotubes. Phys. Chem. C. 115, 24381–24393. 10.1021/jp205052f

[B42] Zhang X. Wang Q. Zou L. H. You J. W. (2016). Facile fabrication of titanium dioxide/fullerene nanocomposite and its enhanced visible photocatalytic activity. J. Colloid Interf. Sci. 466, 56–61. 10.1016/j.jcis.2015.12.013 26706486

[B43] Zhao W. C. Qian D. P. Zhang S. Q. Li S. S. Inganäs O. Gao F.. (2016). Fullerene-free polymer solar cells with over 11% efficiency and excellent thermal stability. Adv. Mater. 28, 4734–4739. 10.1002/adma.201600281 27061511

[B44] Zhou F. Q. Feng H. Fang Y. F. Sun Q. Qian Z. S. (2016). Phenylsulfonic acid functionalized carbon quantum dots based biosensor for acetylcholinesterase activity monitoring and inhibitor screening. RSC Adv. 6, 105454–105460. 10.1039/C6RA18978D

