# Supplementary material for: Fullerol Nanocatalysis and Trimodal Surface Plasmon Resonance for the Determination of Isocarbophos
Source: Front Chem. 2020 Aug 14;8:673. doi: 10.3389/fchem.2020.00673 (PMC7456962; doi:10.3389/fchem.2020.00673)
Supplement: Supplementary file 1 [file Data_Sheet_1.PDF]

Supplementary Material

1 Supplementary Figures and Tables

1.1 Supplementary Figures

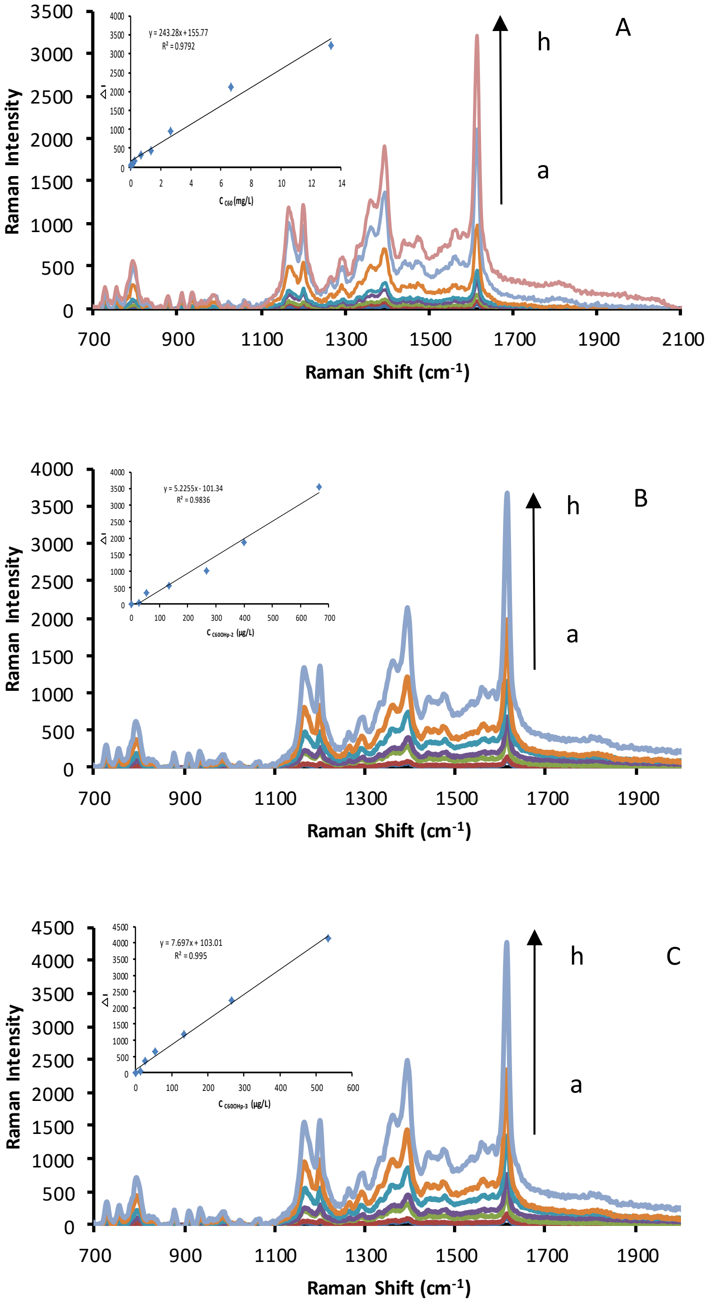

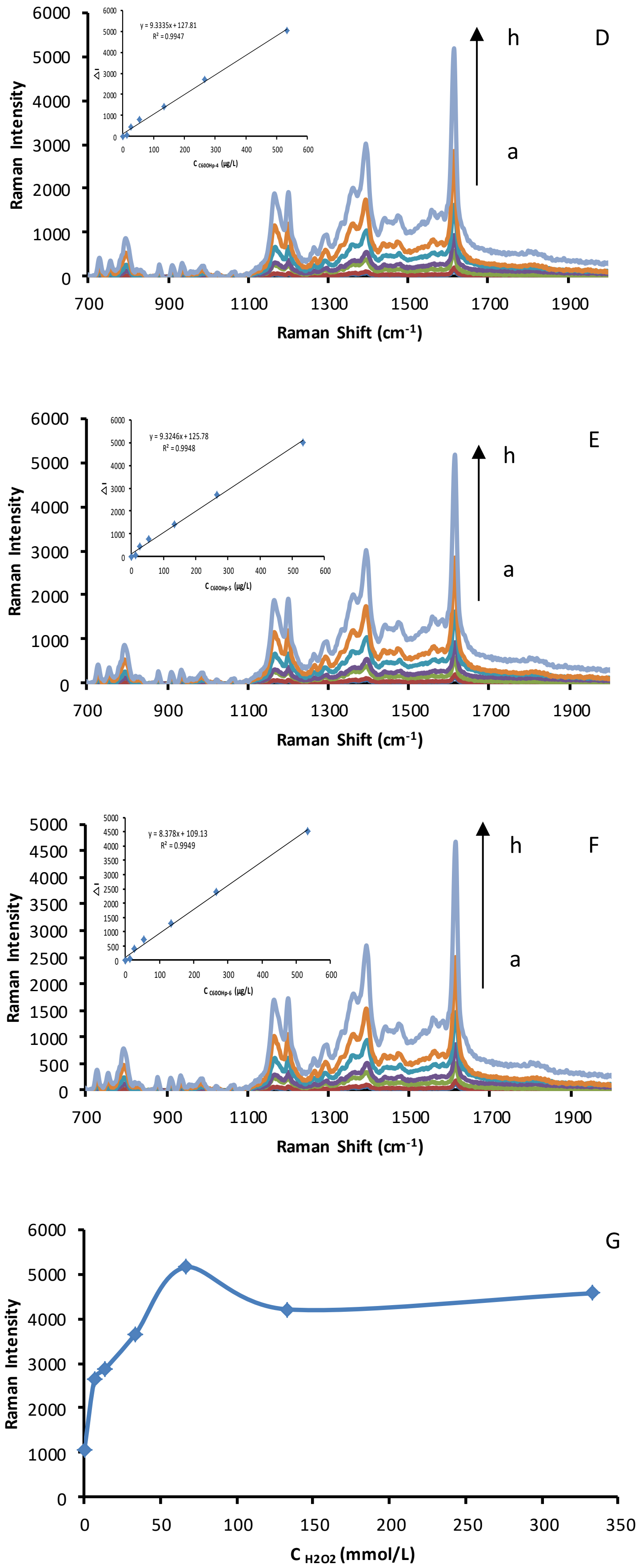

D
h
a
y = 9.3335x + 127.81
R² = 0.9947
Raman Intensity
Raman Shift (cm⁻¹)
E
h
a
y = 9.3246x + 125.78
R² = 0.9948
Raman Intensity
Raman Shift (cm⁻¹)
F
h
a
y = 8.378x + 109.13
R² = 0.9949
Raman Intensity
Raman Shift (cm⁻¹)
G
Raman Intensity
C H2O2 (mmol/L)

Supplementary Figure S1. SERS spectra of $C_{60}OH_P$ -$AgNO_3$-TSC system

0-533.33 μg/L $C_{60}OH_P$ (A: $C_{60}OH_{P-1}$ B: $C_{60}OH_{P-2}$ C: $C_{60}OH_{P-3}$ D: $C_{60}OH_{P-4}$ E: $C_{60}OH_{P-5}$ F: $C_{60}OH_{P-6}$) + 1.33 mmol/L $AgNO_3$ + 4.67 mmol/L TSC +85°C+21min + 3.33×10^{-7} mol/L VBB +0.02 mol/L NaCl.

G: Influence of H_2O_2 on $C_{60}OH_P$ catalytic activity.

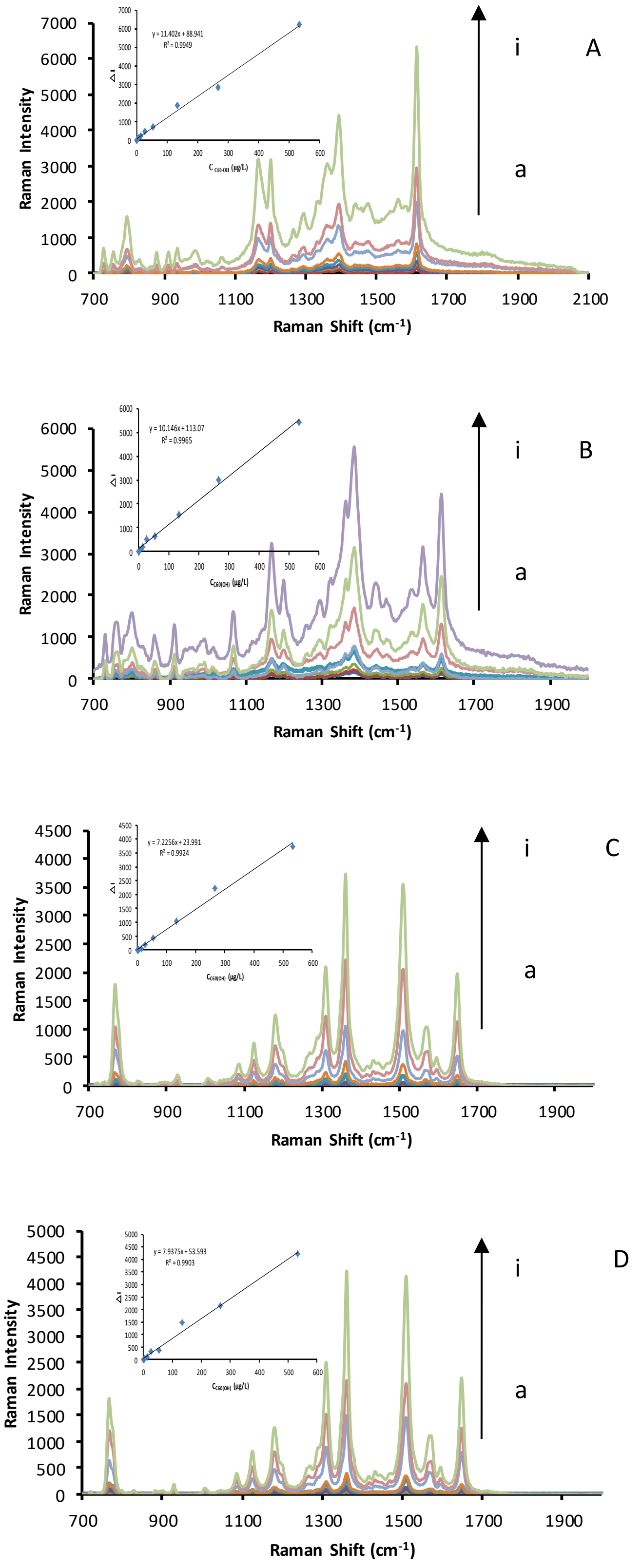

A
B
C
D
i
a
Raman Intensity
Raman Shift (cm-1)
y = 11.402x + 88.941
R² = 0.9949
y = 10.146x + 113.07
R² = 0.9965
y = 7.2256x + 23.991
R² = 0.9924
y = 7.9375x + 53.593
R² = 0.9903

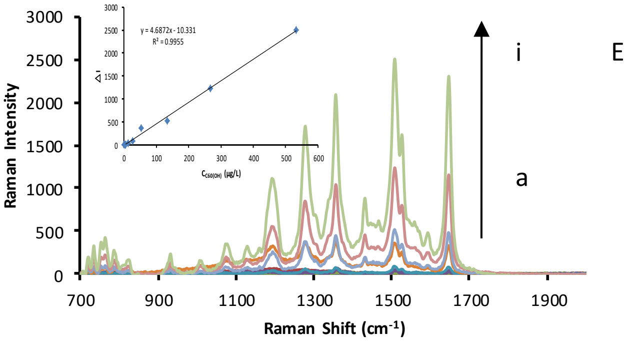

Supplementary Figure S2. SERS spectra of C_{60}OH-$AgNO_3$-TSC system

A： (0, 2.67, 5.33, 13.33, 26.67, 53.33, 133.33, 266.67, 533.33 μg/L) C_{60}OH+ 1.33 mmol/L $AgNO_3$+ 4.67 mmol/L TSC +85℃+21min +3.33×10^{-7} mol/L VBB+0.02 mol/L NaCl

B：(0, 2.67, 5.33, 13.33, 26.67, 53.33, 133.33, 266.67, 533.33 μg/L) C_{60}OH + 1.33 mmol/L $AgNO_3$+ 4.67 mmol/L TSC +85℃+21min +1×10^{-6} mol/L VB4R+0.02 mol/L NaCl

C：(0, 2.67, 5.33, 13.33, 26.67, 53.33, 133.33, 266.67, 533.33 μg/L) C_{60}OH + 1.33 mmol/L $AgNO_3$+ 4.67 mmol/L TSC +85℃+21min +1.67×10^{-6} mol/L RhS+0.02 mol/L NaCl

D：(0, 2.67, 5.33, 13.33, 26.67, 53.33, 133.33, 266.67, 533.33 μg/L) C_{60}OH + 1.33 mmol/L $AgNO_3$+ 4.67 mmol/L TSC +85℃+21min +1×10^{-6} mol/L Rh6G+0.02 mol/L NaCl

E：(0, 2.67, 5.33, 13.33, 26.67, 53.33, 133.33, 266.67, 533.33 μg/L) C_{60}OH + 1.33 mmol/L $AgNO_3$+ 4.67 mmol/L TSC +85℃+21min +1×10^{-5} mol/L RhB+0.02 mol/L NaCl

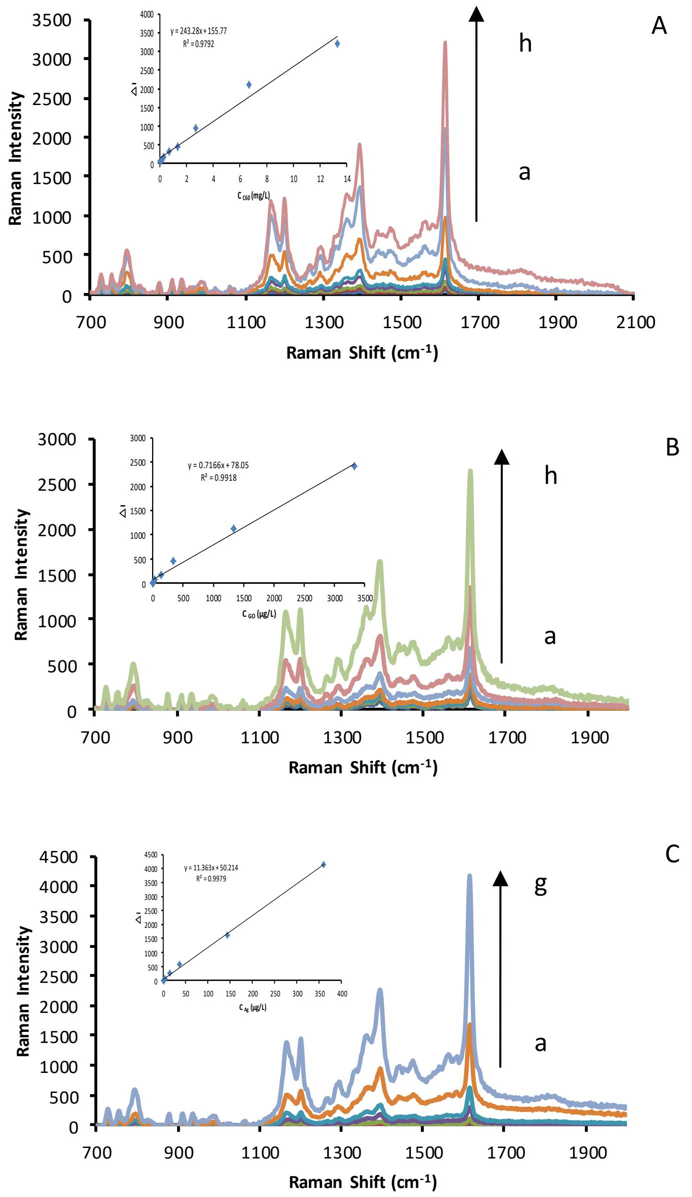

Supplementary Figure S3. SERS spectra of various catalytic-$AgNO_3$-TSC system

A: (0, 0.13, 0.27, 0.67, 1.33, 2.67, 6.67, 13.33 mg/L) C_{60} + 1.33 mmol/L $AgNO_3$+ 4.67 mmol/L TSC +85°C+21min +3.33×10^{-7} mol/L VBB+0.02 mol/L NaCl
B: (0, 2.67, 5.33, 13.33, 26.67, 53.33, 133.33, 266.67, 666.67 μg/L) GO+ 1.33 mmol/L $AgNO_3$+ 4.67 mmol/L TSC +85°C+21min +3.33×10^{-7} mol/L VBB+0.02 mol/L NaCl
C: (0, 1.44, 3.6, 14.38, 35.96, 143.82, 359.56 μg/L) AgNP+ 1.33 mmol/L $AgNO_3$+ 4.67 mmol/L TSC +85°C+21min +3.33×10^{-7} mol/L VBB+0.02 mol/L NaCl

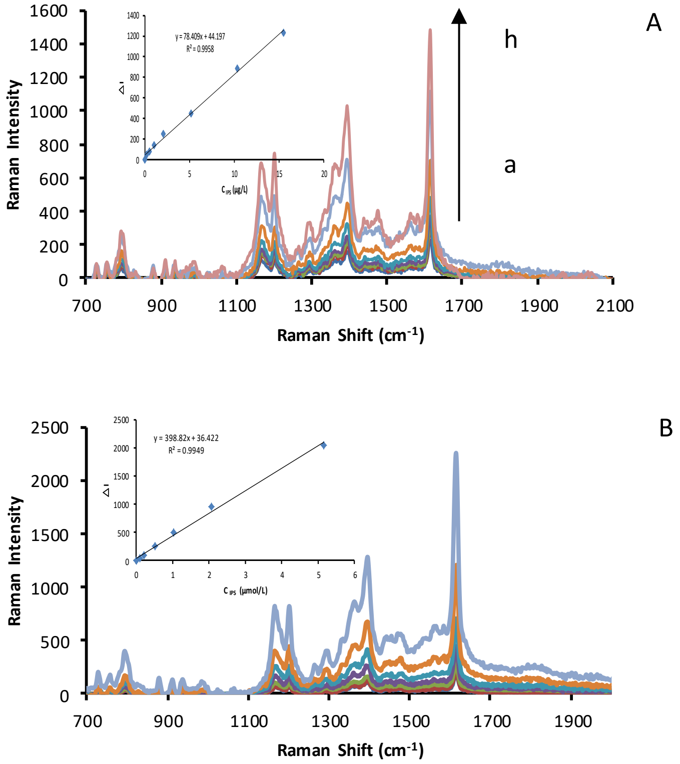

Supplementary Figure S4. SERS spectra of Apt-various catalytic-$AgNO_3$-TSC-IPS system

A: 51.67 nmol/L Apt + 2.67 mg/L C_{60}+ (0, 0.21, 0.52, 1.03, 2.07, 5.17, 10.33, 15.5 μg/L) IPS + 1.33 mmol/L $AgNO_3$+ 4.67 mmol/L TSC +85°C+21min +3.33×10^{-7} mol/L VBB+0.02 mol/L NaCl

B: 20.67 nmol/L Apt + 266.67 μg/L $C_{60}OH_P$+ (0, 0.05, 0.21, 0.52, 2.07, 5.17μg/L) IPS + 1.33 mmol/L $AgNO_3$+ 4.67 mmol/L TSC +85°C+21min +3.33×10^{-7} mol/L VBB+0.02 mol/L NaCl

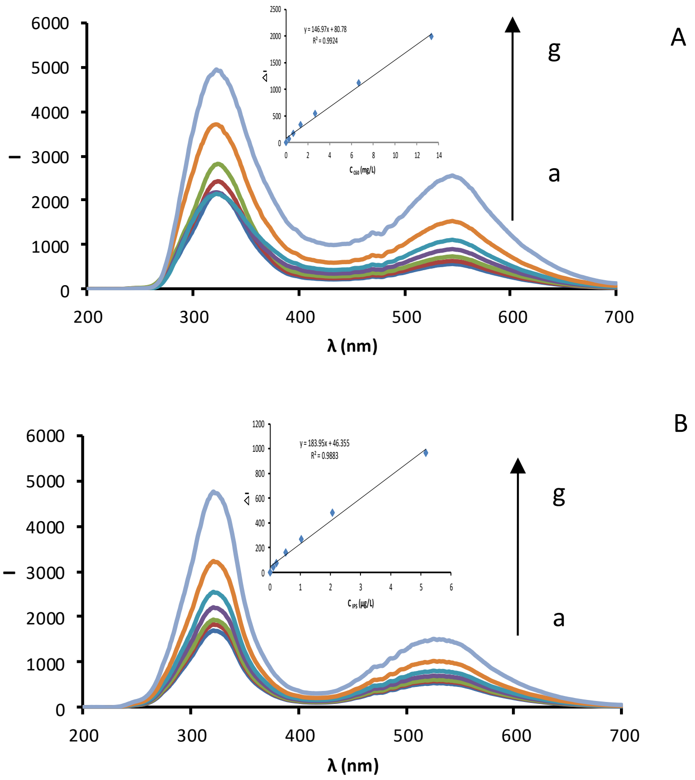

Supplementary Figure S5. RRS spectra of C_{60} system

A: (0, 0.27, 0.67, 1.33, 2.67, 6.67, 13.33 mg/L) C_{60}+ 1.33 mmol/L $AgNO_3$+ 4.67 mmol/L TSC +85℃+21min +3.33×10^{-7} mol/L VBB+0.02 mol/L NaCl

B: 51.67 nmol/L Apt + 2.67 mg/L C_{60}+ (0, 0.1, 0.21, 0.52, 1.03, 2.07, 5.17 μg/L) IPS + 1.33 mmol/L $AgNO_3$+ 4.67 mmol/L TSC +85℃+21min +3.33×10^{-7} mol/L VBB+0.02 mol/L NaCl

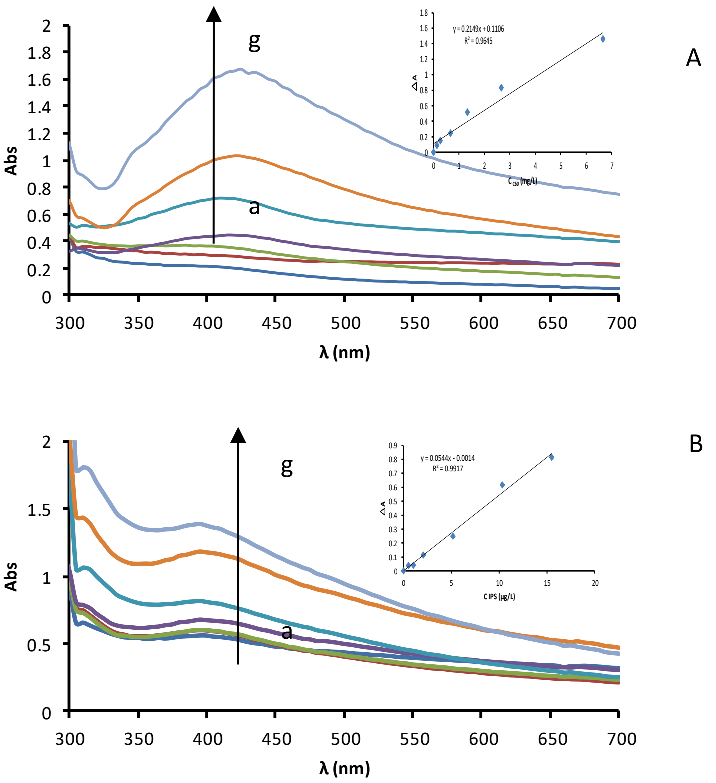

Supplementary Figure S6. Absorption spectra of C_{60}- $AgNO_3$-TSC system

A: (0, 0.13, 0.27, 0.67, 1.33, 2.67, 6.67 mg/L) C_{60}+ 1.33 mmol/L $AgNO_3$ + 4.67 mmol/L TSC +85°C+21min +3.33×10^{-7} mol/L VBB+0.02 mol/L NaCl

B: 51.67 nmol/L Apt + 2.67 mg/L C_{60}+ (0, 0.52, 1.03, 2.07, 5.17, 10.33, 15.5 μg/L) IPS + 1.33 mmol/L $AgNO_3$+ 4.67 mmol/L TSC +85°C+21min +3.33×10^{-7} mol/L VBB+0.02 mol/L NaCl

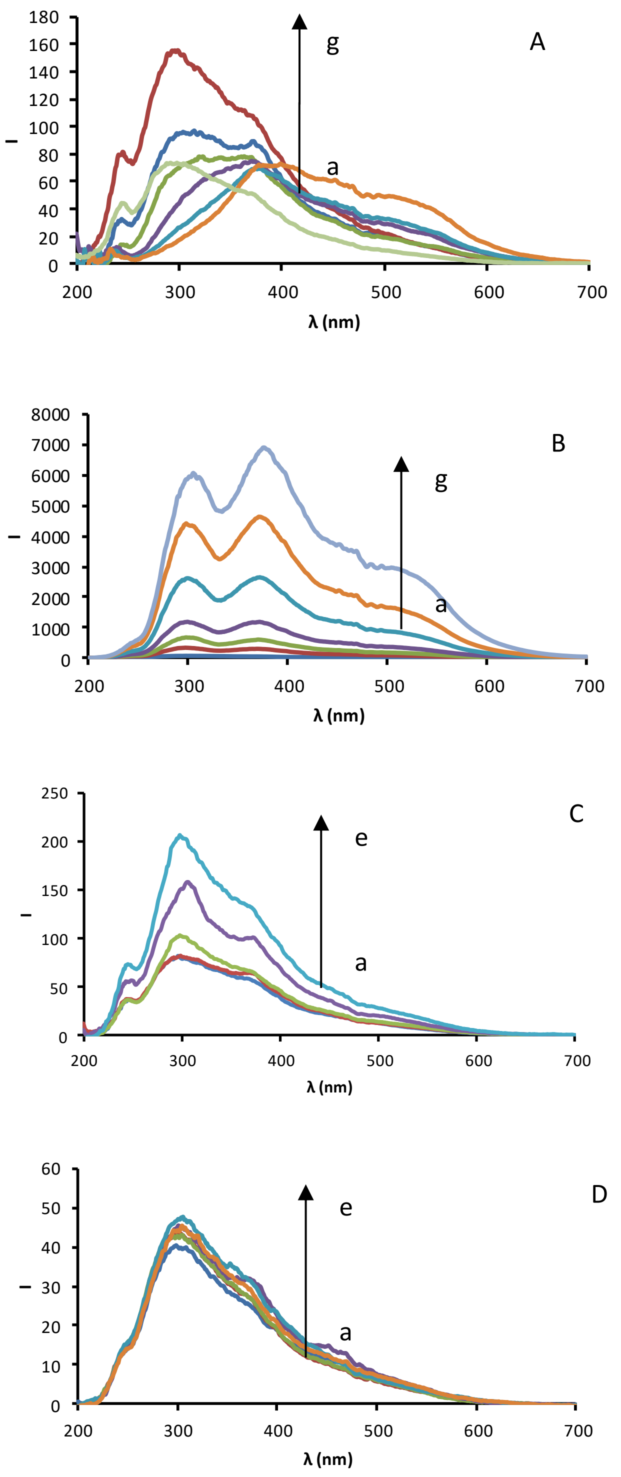

Supplementary Figure S7. RRS spectra of $C_{60}OH$ and C_{60}

A：（0, 0.13, 0.27, 0.53, 1.33, 2.67, 5.33 mg/L）$C_{60}OH$

B：（0, 0.5, 1, 2.5, 5, 10, 20 mg/L）C_{60}

C： 0.27 mg/L $C_{60}OH$+（0, 1.03, 5.17, 51.7, 103.3nmol/L）Apt;

D： 0, 1.03, 5.17, 51.7, 103.3nmol/L Apt

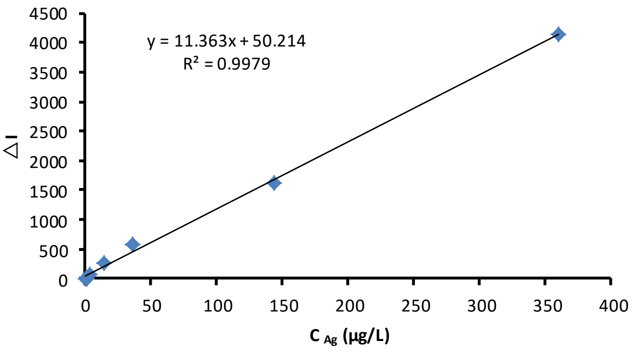

Supplementary Figure S8. The SERS linear equation of AgNP-$AgNO_3$-TSC system

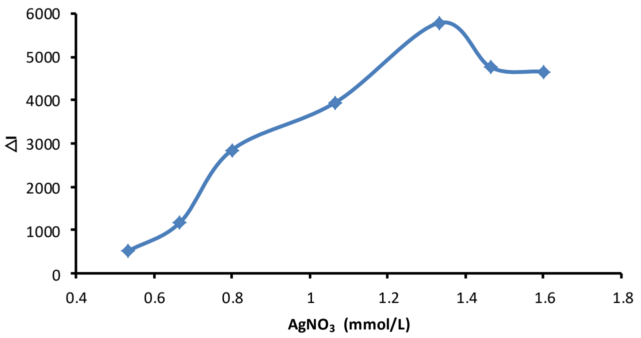

Supplementary Figure S9. Effect of $AgNO_3$ concentration on the △I value

266.67 μg/L $C_{60}OH$ +x mmol/L $AgNO_3$ +6.67 mmol/L TSC+85℃+ 21 min +3.33×10^{-7} mol/L VBB+0.02 mol/L NaCl

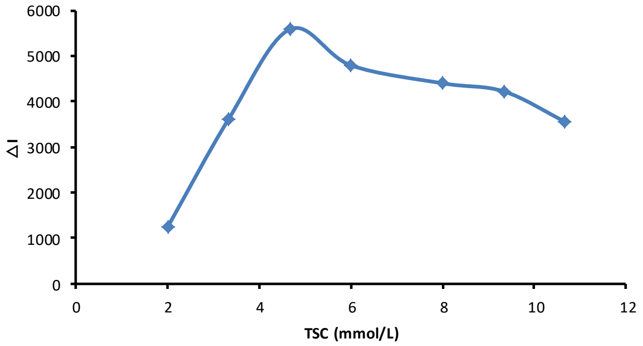

Supplementary Figure S10. Effect of TSC concentration on the △I value

266.67 μg/L $C_{60}OH$ + 1.33 mmol/L $AgNO_3$ +x mmol/L TSC +85℃+ 21 min +3.33×10^{-7} mol/L VBB+0.02 mol/L NaCl

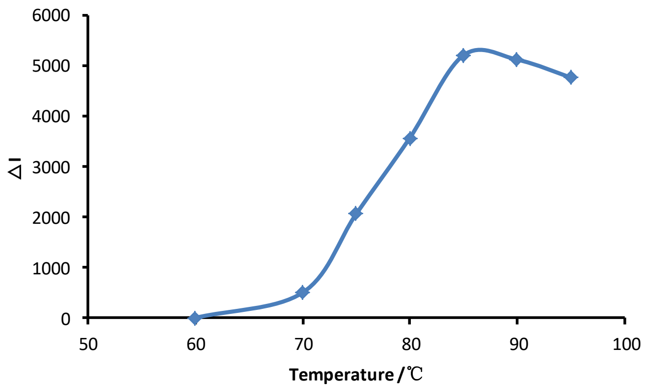

Supplementary Figure S11. Effect of temperature on the △I value

266.67 μg/L $C_{60}OH$ + 1.33 mmol/L $AgNO_3$ +4.67 mmol/L TSC + x ℃+ 21 min +3.33×10^{-7} mol/L VBB+0.02 mol/L NaCl

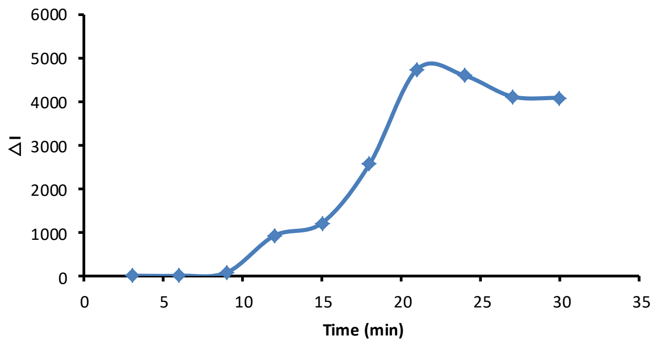

Supplementary Figure S12. Effect of time on the △I value

266.67 μg/L $C_{60}OH$+ 1.33 mmol/L $AgNO_3$+ 4.67 mmol/L TSC+85℃+ x min +3.33×10^{-7} mol/L VBB+0.02 mol/L NaCl

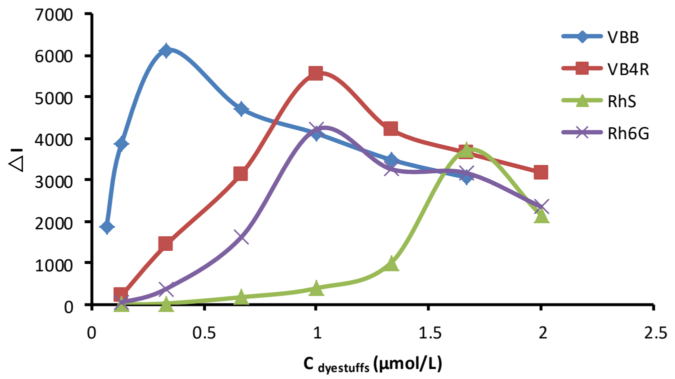

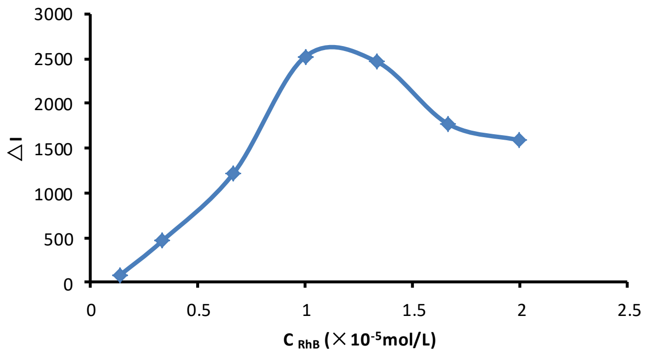

Supplementary Figure S13. Effect of various dyestuffs on the △I value

266.67 μg/L $C_{60}OH$+ 1.33 mmol/L $AgNO_3$+ 4.67 mmol/L TSC +85℃+ 21 min + various dyestuffs +0.02 mol/L NaCl

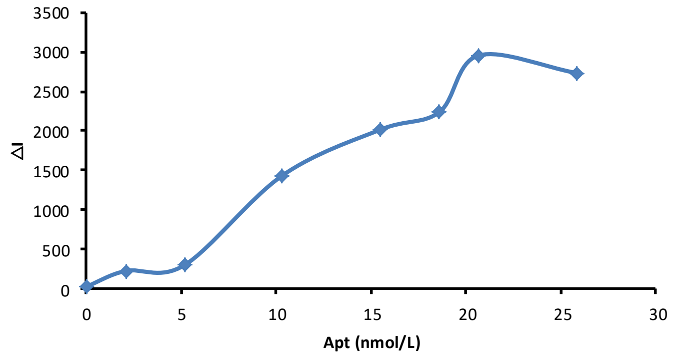

Supplementary Figure S14. Effect of Apt on the △I value

x nmol/L Apt + 5 µg/LIPS +266.67 µg/L $C_{60}OH$ + 1.33 mmol/L $AgNO_3$+ 4.67 mmol/L TSC +85℃+ 21 min +3.33×10^{-7} mol/L VBB+0.02 mol/L NaCl

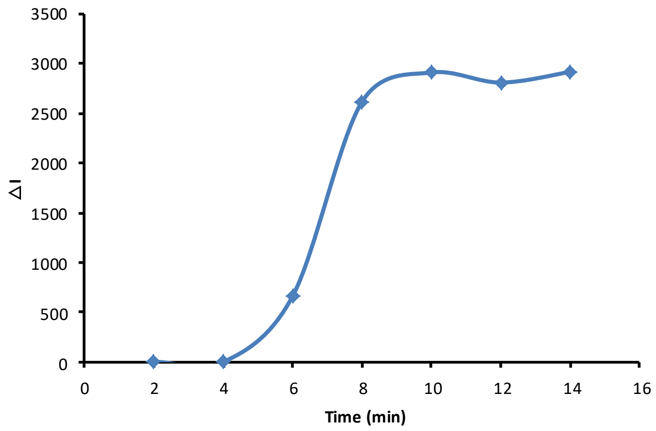

Supplementary Figure S15. Effect of binding time on the △I value

20.67 nmol/L Apt + 5 μg/LIPS+266.67 μg/L $C_{60}OH$ + 1.33 mmol/L $AgNO_3$+ 4.67 mmol/L TSC +85°C+ 21 min +3.33×10^{-7} mol/L VBB+0.02 mol/L NaCl

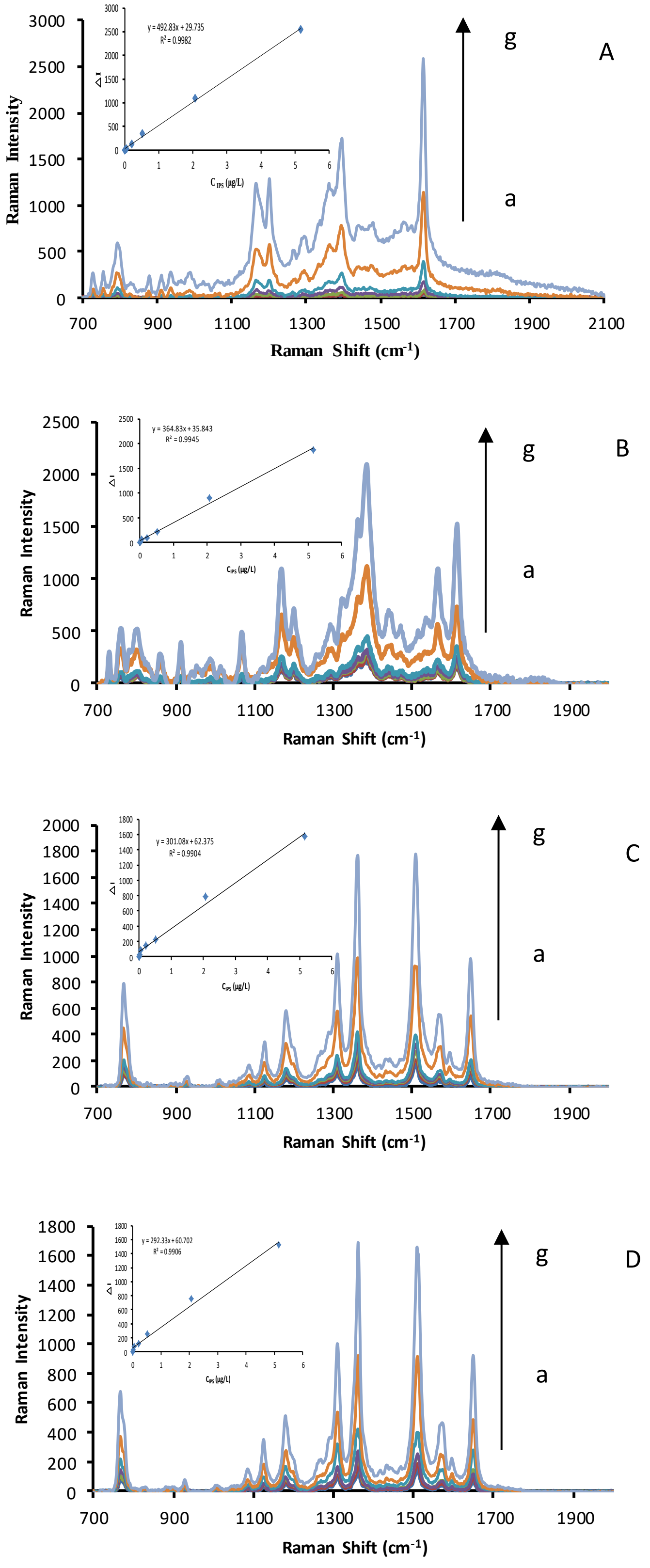
A
B
C
D
Raman Intensity
Raman Shift (cm-1)
g
a
y = 492.83x + 29.735
R² = 0.9982
y = 364.83x + 35.843
R² = 0.9945
y = 301.08x + 62.375
R² = 0.9904
y = 292.33x + 60.702
R² = 0.9906
C IPS (μg/L)
△I

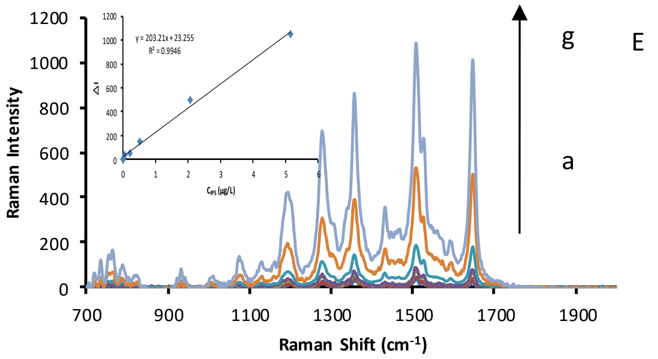

Supplementary Figure S16. SERS spectra of Apt-C_{60}OH-$AgNO_3$-TSC-IPS system

A：20.67 nmol/L Apt + 266.67 μg/L C_{60}OH+ (0, 0.02, 0.05, 0.21, 0.52, 2.07, 5.17μg/L) IPS + 1.33 mmol/L $AgNO_3$+ 4.67 mmol/L TSC+85℃+21min +3.33×10^{-7} mol/L VBB+0.02 mol/L NaCl

B：20.67 nmol/L Apt + 266.67 μg/L C_{60}OH+ (0, 0.02, 0.05, 0.21, 0.52, 2.07, 5.17μg/L) IPS + 1.33 mmol/L $AgNO_3$+ 4.67 mmol/L TSC +85℃+21min +1×10^{-6} mol/L VB4R +0.02 mol/L NaCl

C：20.67 nmol/L Apt + 266.67 μg/L C_{60}OH+ (0, 0.02, 0.05, 0.21, 0.52, 2.07, 5.17μg/L) IPS + 1.33 mmol/L $AgNO_3$+ 4.67 mmol/L TSC +85℃+21min +1.67×10^{-6} mol/L RhS +0.02 mol/L NaCl

D：20.67 nmol/L Apt + 266.67 μg/L C_{60}OH+ (0, 0.02, 0.05, 0.21, 0.52, 2.07, 5.17μg/L) IPS + 1.33 mmol/L $AgNO_3$+ 4.67 mmol/L TSC +85℃+21min +1×10^{-6} mol/L Rh6G +0.02 mol/L NaCl

E：20.67 nmol/L Apt + 266.67 μg/L C_{60}OH+ (0, 0.02, 0.05, 0.21, 0.52, 2.07, 5.17μg/L) IPS + 1.33 mmol/L $AgNO_3$+ 4.67 mmol/L TSC +85℃+21min +1×10^{-5} mol/L RhB +0.02 mol/L NaCl

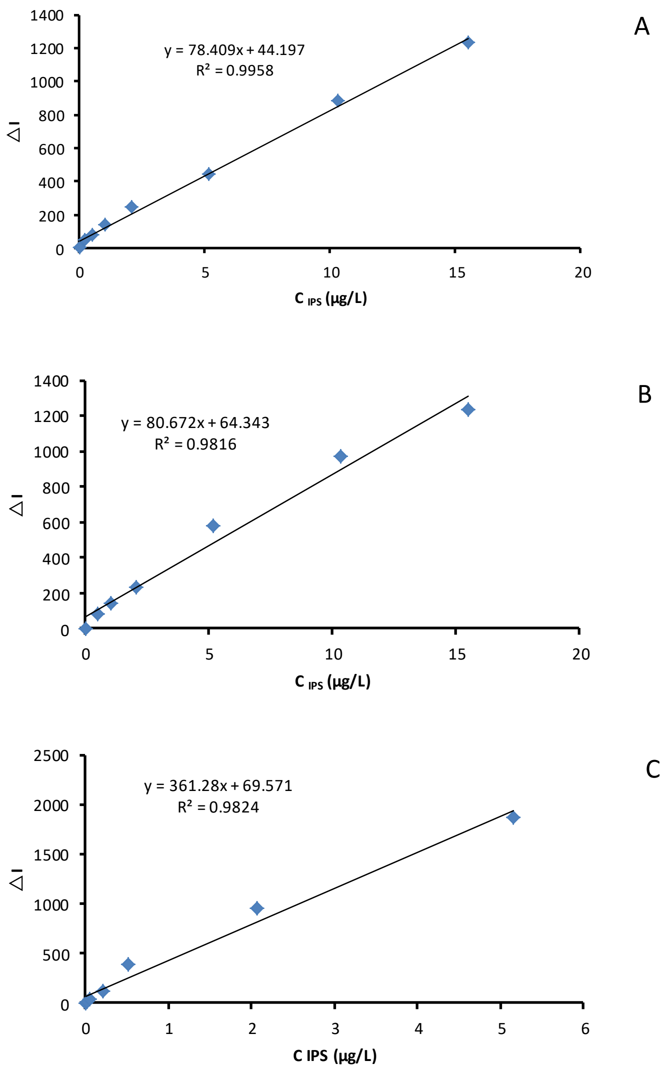

Supplementary Figure S17. Working curve for the SERS determination of Apt-various catalytic- $AgNO_3$-TSC-IPS

A: 51.67 nmol/L Apt + 2.67 mg/L C_{60}+ (0.21-15.5 µg/L) IPS + 1.33 mmol/L $AgNO_3$+ 4.67 mmol/L TSC+85℃+21min +3.33×10^{-7} mol/L VBB+0.02 mol/L NaCl
B: 51.67 nmol/L Apt + 1333.33 µg/L GO + 0.05-5µg /L IPS + 1.33 mmol/L $AgNO_3$+ 4.67 mmol/L TSC+85℃+ 21 min +3.33×10^{-7} mol/L VBB+0.02 mol/L NaCl
C: 20.67 nmol/L Apt + 0.05-5µg /L IPS + 143.82 µg/L AgNP + 1.33 mmol/L $AgNO_3$+ 4.67 mmol/L TSC+85℃+ 21 min +3.33×10^{-7} mol/L VBB+0.02 mol/L NaCl

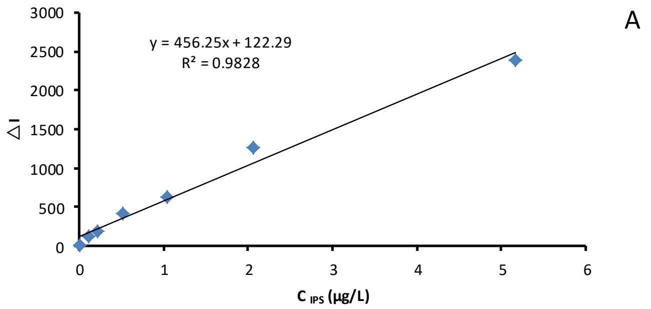

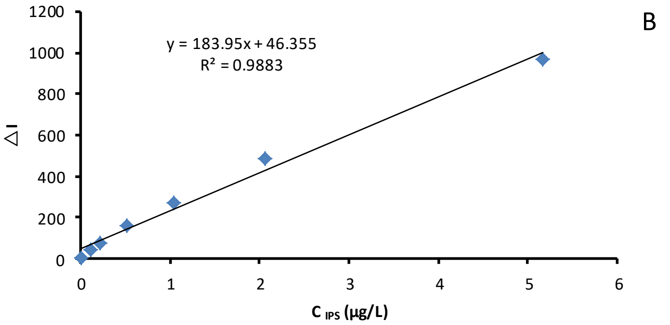

Supplementary Figure S18. Working curve for the RRS determination of Apt-various catalytic- $AgNO_3$-TSC-IPS

A: 20.67 nmol/L Apt + 266.67 μg/L $C_{60}OH$ + (0, 0.1, 0.21, 0.52, 1.03, 2.07, 5.17μg/L) IPS + 1.33 mmol/L $AgNO_3$+ 4.67 mmol/L TSC+85℃+21min +3.33×10^{-7} mol/L VBB+0.02 mol/L NaCl

B: 51.67 nmol/L Apt + 2.67 mg/L C_{60}+ (0, 0.1, 0.21, 0.52, 1.03, 2.07, 5.17 μg/L) IPS + 1.33 mmol/L $AgNO_3$+ 4.67 mmol/L TSC+85℃+21min +3.33×10^{-7} mol/L VBB+0.02 mol/L NaCl

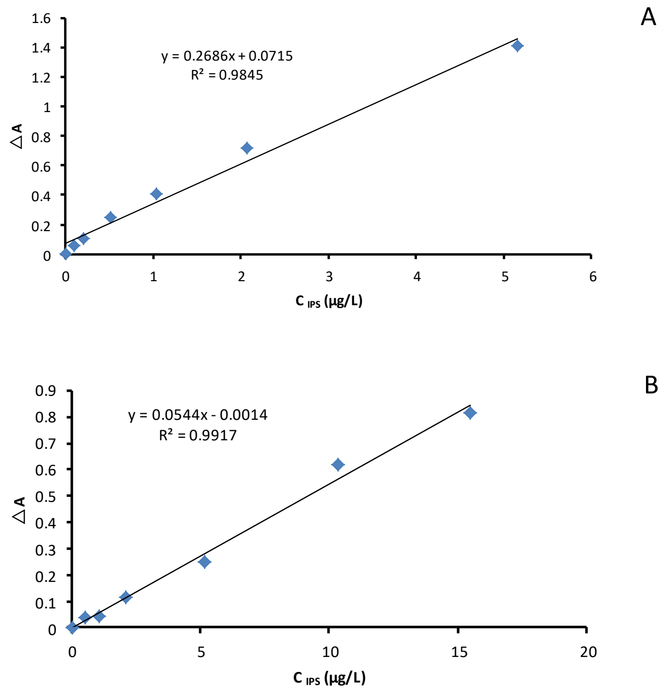

Supplementary Figure S19. Working curve for the Abs determination of Apt-various catalytic- $AgNO_3$-TSC-IPS

A: 20.67 nmol/L Apt + 266.67 μg/L C_{60}OH + (0, 0.1, 0.21, 0.52, 1.03, 2.07, 5.17μg/L) IPS + 1.33 mmol/L $AgNO_3$+ 4.67 mmol/L TSC+85°C+21min +3.33×10^{-7} mol/L VBB+0.02 mol/L NaCl

B: 51.67 nmol/L Apt + 2.67 mg/L C_{60}+ (0, 0.52, 1.03, 2.07, 5.17, 10.33, 15.5 μg/L) IPS + 1.33 mmol/L $AgNO_3$+ 4.67 mmol/L TSC+85°C+21min +3.33×10^{-7} mol/L VBB+0.02 mol/L NaCl

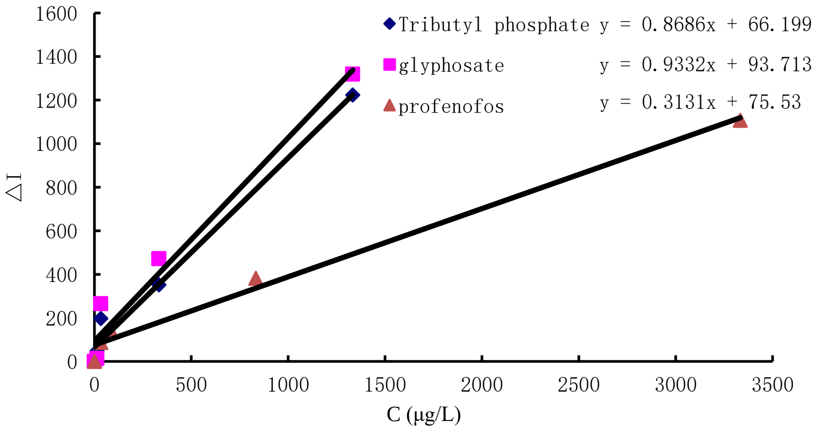

Supplementary Figure S20. Analytical characteristics for the SERS determination of related organic phosphorus

20.67 nmol/L Apt + organic phosphorus + 266.67 μg/L $C_{60}OH$ + 1.33 mmol/L $AgNO_3$+ 4.67 mmol/L TSC+85℃+ 21 min +3.33×10^{-7} mol/L VBB+0.02 mol/L NaCl

1.2 Supplementary Tables

Supplementary Table S1 Comparison of reported analytical methods for IPS

Method	Principle	Linearly range	Limit of detection	Annotation	Reference
Spectrophotometry	IPS-aptamer-hemin precipitin reaction result in the absorbency decreased.	0.4-40 μg/L	0.2 μg/L	Accuracy, low sensitivity	37
Capillary electrophoresis	The ionic liquids ([BMIM]BF_4) and SBS used as separating agent. CE-GC-flame photometer for IPS detection.		0.08mg/kg	Environment friendly, low sensitivity	33
Fluorimetric method	Complement aptamer and IPS competitive Combination with Apt-MB that turn on-off system fluorescence.		5 μg/L	Rapid, simple, low sensitivity	38
Chemiluminescence	IPS catalyzed H_2O_2-luminol to enhance system chemiluminescence.	0.086–15ug/ml	0.05ug/ml	Sensitivity, expensive equipment, complexity.	32
Electrochemical method	Molecularly imprinted terpolymer modified on a glassy carbon electrode to selective electrochemistry for IPS detection.	7.5×10^{-8} — 1.00×10^{-4}mol/L	2.01×10^{-8} mol/L	Selectivity, convenience, low sensitivity.	36
SERS	IPS combined with Ag-Apt-MH and quenched its SERS effect.		1mg/L	Rapid, selectivity, low sensitivity.	39
This method	Apt adjusted nanocatalyst activity to generate various amount AgNP. SERS, RRS method for IPS detection.	0.02~5.17 μg/L	8.2 ng/L	Sensitivity, selectivity, simple.	

Supplementary Table S2 Selectivity of the analysis of IPS by the SERS method

coexistent ion	Limit times	coexistent ion	Limit times	coexistent ion	Limit times
Glyphosate	80	K^{+}	400	Al^{3+}	150
Profenofos	120	Zn^{2+}	500	Fe^{3+}	200
Tributyl phosphine	200	Mg^{2+}	300	Ni^{2+}	80
CO_3^{2-}	1200	NO_3^{-}	400	Ba^{2+}	350
Na^{+}	400	Ca^{2+}	500	Fe^{2+}	100
HCO_3^{-}	600	Cu^{2+}	100	Bi^{2+}	240
Cl^{-}	400	NH_4^{+}	400		
